# A series of tetraazalene radical-bridged M_2_ (M = Cr^III^, Mn^II^, Fe^II^, Co^II^) complexes with strong magnetic exchange coupling†
†Electronic supplementary information (ESI) available: Experimental details, UV/Vis/NIR spectra for **2–8**, additional magnetic data for **4–8**, crystallographic data, selected bond distances, and crystallographic information files (CIFs) for **1**, **2**·0.4THF, **3**·2.5THF, **4**·2.5THF, and **5**·2.9MeCN (CCDC 1414648–1414652). For ESI and crystallographic data in CIF or other electronic format see DOI: 10.1039/c5sc02725j


**DOI:** 10.1039/c5sc02725j

**Published:** 2015-08-18

**Authors:** Jordan A. DeGayner, Ie-Rang Jeon, T. David Harris

**Affiliations:** a Department of Chemistry , Northwestern University , 2145 Sheridan Road , Evanston , IL , USA 60208-3113 . Email: dharris@northwestern.edu

## Abstract

The ability of tetraazalene radical bridging ligands to mediate exceptionally strong magnetic exchange coupling across a range of transition metal complexes is demonstrated. The redox-active bridging ligand *N*,*N*′,*N*′′,*N*′′′-tetra(2-methylphenyl)-2,5-diamino-1,4-diiminobenzoquinone (^NMePh^LH_2_) was metalated to give the series of dinuclear complexes [(TPyA)_2_M_2_(^NMePh^L^2–^)]^2+^ (TPyA = tris(2-pyridylmethyl)amine, M = Mn^II^, Fe^II^, Co^II^). Variable-temperature dc magnetic susceptibility data for these complexes reveal the presence of weak superexchange interactions between metal centers, and fits to the data provide coupling constants of *J* = –1.64(1) and –2.16(2) cm^–1^ for M = Mn^II^ and Fe^II^, respectively. One-electron reduction of the complexes affords the reduced analogues [(TPyA)_2_M_2_(^NMePh^L^3–^˙)]^+^. Following a slightly different synthetic procedure, the related complex [(TPyA)_2_CrIII2(^NMePh^L^3–^˙)]^3+^ was obtained. X-ray diffraction, cyclic voltammetry, and Mössbauer spectroscopy indicate the presence of radical ^NMePh^L^3–^˙ bridging ligands in these complexes. Variable-temperature dc magnetic susceptibility data of the radical-bridged species reveal the presence of strong magnetic interactions between metal centers and ligand radicals, with simulations to data providing exchange constants of *J* = –626(7), –157(7), –307(9), and –396(16) cm^–1^ for M = Cr^III^, Mn^II^, Fe^II^, and Co^II^, respectively. Moreover, the strength of magnetic exchange in the radical-bridged complexes increases linearly with decreasing M–L bond distance in the oxidized analogues. Finally, ac magnetic susceptibility measurements reveal that [(TPyA)_2_Fe_2_(^NMePh^L^3–^˙)]^+^ behaves as a single-molecule magnet with a relaxation barrier of *U*_eff_ = 52(1) cm^–1^. These results highlight the ability of redox-active tetraazalene bridging ligands to enable dramatic enhancement of magnetic exchange coupling upon redox chemistry and provide a rare opportunity to examine metal–radical coupling trends across a transmetallic series of complexes.

## Abstract 2

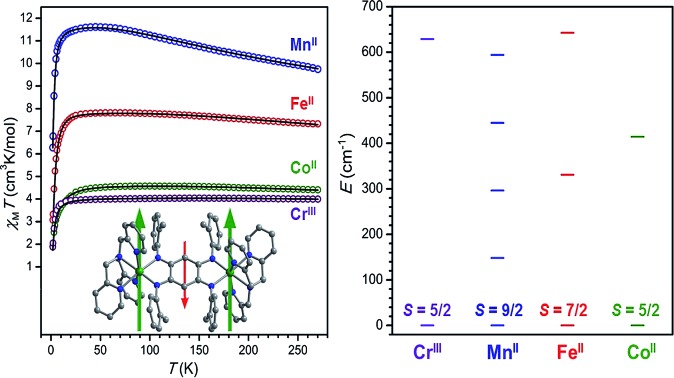
The ability of tetraazalene radical bridging ligands to mediate exceptionally strong magnetic exchange coupling across a range of transition metal complexes is demonstrated.

## Introduction

Over the past two decades, a number of coordination compounds, from mono- and multinuclear metal complexes^1^ to extended solids,^2^–^4^ have been shown to display magnetic bistability. Such molecule-based magnets are commonly constructed through judicious selection of bridging ligand and paramagnetic metal ions, thereby enabling the rational design and direct synthetic control for optimizing magnetic properties. In addition to these synthetic advantages, there is tremendous interest to develop molecule-based magnetic materials for potential use in applications such as high-density information storage, permanent magnet design, magnetic sensing, and gas separation,^4^–^6^ owing largely to their small size, low density, and chemical tunability. In order to realize such applications at ambient temperature, magnets displaying ordering or slow dynamics at higher temperature must be synthesized. As such, engendering strong magnetic exchange interactions represents an important challenge in the design of molecule-based magnets, as the strength of magnetic exchange between paramagnetic centers is directly related to the critical temperature of two- or three-dimensional magnets,^7^ the thermal relaxation barrier of single-chain magnets,^8^ and the isolation of the spin ground state of single-molecule magnets.^9^

Despite the critical importance of coupling strength in magnetic materials, the vast majority of molecule-based magnets feature structures comprised of paramagnetic metal ions bridged by diamagnetic ligands. Here, the mechanism of magnetic exchange between metal ions is indirect superexchange through the ligand, leading to relatively weak magnetic coupling, particularly when involving multi-atom bridging ligands. While bridging ligands that feature only one or two atoms, such as oxo and cyano ligands, can in some cases mediate sufficiently strong superexchange to give room-temperature three-dimensional magnets,3f–i their chemistry is limited by the inherent lack of structural diversity and possible ligand functionalization. Alternatively, incorporation of a paramagnetic bridging ligand can give rise to much stronger magnetic exchange coupling owing to the direct overlap of orbitals that contain unpaired electrons.^10^ Indeed, this strategy has been central in the realization of the first room-temperature molecule-based magnet,3*c* the first experimental observation of slow dynamics in one-dimensional chain compounds,2*a* and the highest magnetic blocking temperature of any single-molecule magnet.1*l*

**Scheme 1:**
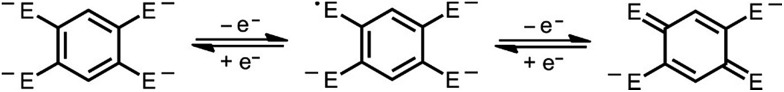
Redox series of deprotonated benzoquinonoid ligands. Left to right: ^E^L^4–^, ^E^L^3–^˙, ^E^L^2–^ (E = O and NR).

Considering the goal of installing strong magnetic exchange between a metal ion and a paramagnetic ligand, quinonoid-type ligands offer an ideal platform for the construction of molecule-based magnets,^11^ as these molecules readily accommodate redox chemistry to stabilize both diamagnetic and paramagnetic redox isomers (see Scheme 1). Indeed, a number of dinuclear complexes bridged by tetraoxolene radicals have been shown to exhibit strong metal–ligand interactions.^12^ Furthermore, the strength of magnetic exchange through dia- and paramagnetic quinonoid ligands has been found to be even stronger upon moving to nitrogen donors, owing to their more diffuse orbitals compared to oxygen.^13^ Recently, we reported a dinuclear azophenine radical-bridged FeII2 single-molecule magnet which features an *S* = 7/2 ground state that remains well-isolated even at 300 K, with an estimated coupling constant of *J* ≤ –900 cm^–1^.^14^ This observation, in conjunction with the dearth of radical-bridged molecule-based magnets featuring strong magnetic exchange, prompted us to extend this work to other transition metal ions, both to assess the generalizability of this approach and also to elucidate the role structure plays in governing metal–ligand radical interactions. Indeed, while such systematic studies are rare,^15^ they are nonetheless critical to uncover the fundamental magnetostructural correlations needed to inform the design of new magnetic materials that function at high temperature.

Herein, we report the synthesis and detailed characterization of the series of dinuclear complexes [(TPyA)_2_M_2_(^NMePh^L^2–^)]^2+^ (M = Mn^II^, Fe^II^, Co^II^) and the radical-bridged analogues [(TPyA)_2_M_2_(^NMePh^L^3–^˙)]^*n*+^ (*n* = 3: M = Cr^III^; *n* = 1: M = Mn^II^, Fe^II^, Co^II^). The magnetic coupling in these complexes exhibits significantly enhanced strength upon ligand radical formation, and the resulting coupling constants are among the largest ever reported for multinuclear complexes. In addition, the magnitude of metal–ligand radical coupling is found to increase linearly with decreasing M–L bond distance in the unreduced analogues, thereby providing a rare example of a magnetostructural correlation in a transmetallic series of metal–radical complexes.

## Results and discussion

### Syntheses and structures

**Scheme 2:**
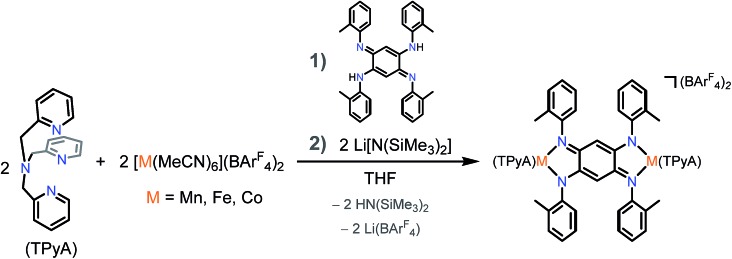
Synthesis of the compounds [(TPyA)_2_MII2(^NMePh^L^2–^)](BArF4)_2_ as observed in **2** (Mn), **3** (Fe), and **4** (Co).

The bridging ligand ^NMePh^LH_2_ was synthesized through the Pd-catalyzed Buchwald–Hartwig amination of 1,2,4,5-tetrabromobenzene with *o*-toluidine, followed by aerobic oxidation, according to a modified literature procedure.^16^ Addition of ^NMePh^LH_2_ to two equivalents each of a solution of TPyA and [M(MeCN)_6_](BArF4)_2_ (M = Mn, Fe, Co) in THF, followed by careful addition of two equivalents of a THF solution of Li[N(SiMe_3_)_2_], afforded a dark-brown solution (see Scheme 2). Careful layering of hexanes onto the resulting THF solutions afforded dark brown, needle-shaped crystals of [(TPyA)_2_MII2(^NMePh^L^2–^)](BArF4)_2_·*x*THF (M = Mn, *x* = 0.4 (**2**·0.4THF), M = Fe, *x* = 2.5 (**3**·2.5THF), M = Co, *x* = 2.5 (**4**·2.5THF)) suitable for single-crystal X-ray diffraction analysis. Subsequent drying of these crystals under reduced pressure gave the desolvated forms in moderate yields of 69%, 50%, and 70% for **2–4**, respectively. In the case of M = Cr, a slightly different procedure was necessary to give crystalline product. Here, ^NMePh^L^2–^ was generated as described above, and metalation was effected through the addition of two equivalents each of a solution of TPyA and [Cr(MeCN)_6_](BArF4)_2_ in THF. The solvent was then removed under reduced pressure, and the resulting powder was washed with hexanes and dried under reduced pressure. This solid was then dissolved in cold MeOH and treated with a MeOH solution containing 6 eq. of Na(BPh_4_) at –78 °C. Filtration of the resulting mixture led to the isolation of a purple solid that was washed with cold MeOH and Et_2_O. Subsequent vapor diffusion of diethyl ether into a MeCN solution of this solid yielded purple, needle-shaped crystals of [(TPyA)_2_CrIII2(^NMePh^L^3–^˙)](BPh_4_)_3_·4.3MeCN (**5**·2.9MeCN), presumably through a spontaneous one-electron oxidation. Drying under reduced pressure gave the partially desolvated product [(TPyA)_2_CrIII2(^NMePh^L^3–^˙)](BPh_4_)_3_·1.4MeCN (**5**) in 27% yield. This compound could be further reacted with a stoichiometric amount of [Cp_2_Fe](BPh_4_) in MeCN to give a dark brown solution. Diffusion of diethyl ether vapor into this solution afforded a mixture of products that included brown, plate-shaped crystals of [(TPyA)_2_Cr_2_(^NMePh^L)](BPh_4_)_4_·4MeCN (**1**). All attempts to obtain this compound in pure bulk form have been unsuccessful, likely owing to dissociation of ^NMePh^L^2–^, as has been previously observed for a tetraoxolene bridged CrIII2 complex.11*b* Nevertheless, to our knowledge, **1–4** represent the first examples of paramagnetic transition metal complexes coordinated by *N*,*N*′,*N*′′,*N*′′′-tetra(2-methylphenyl)-2,5-diamido-1,6-diiminobenzoquinone^17^ and provide a significant expansion of the currently sparse class of dinuclear complexes that incorporate tetraazalene bridging ligands.^18^

**Fig. 1:**
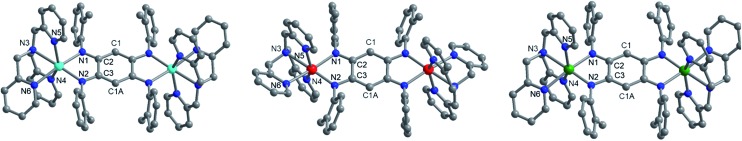
Left–Right: Crystal structures of [(TPyA)_2_MII2(^NMePh^L^2–^)]^2+^ (M = Mn, Fe, Co), as observed in **2**·0.4THF, **3**·2.5THF, and **4**·2.5THF. Cyan, orange, green, blue, and gray spheres represent Mn, Fe, Co, N, and C atoms, respectively; H atoms are omitted for clarity.

**Fig. 2:**
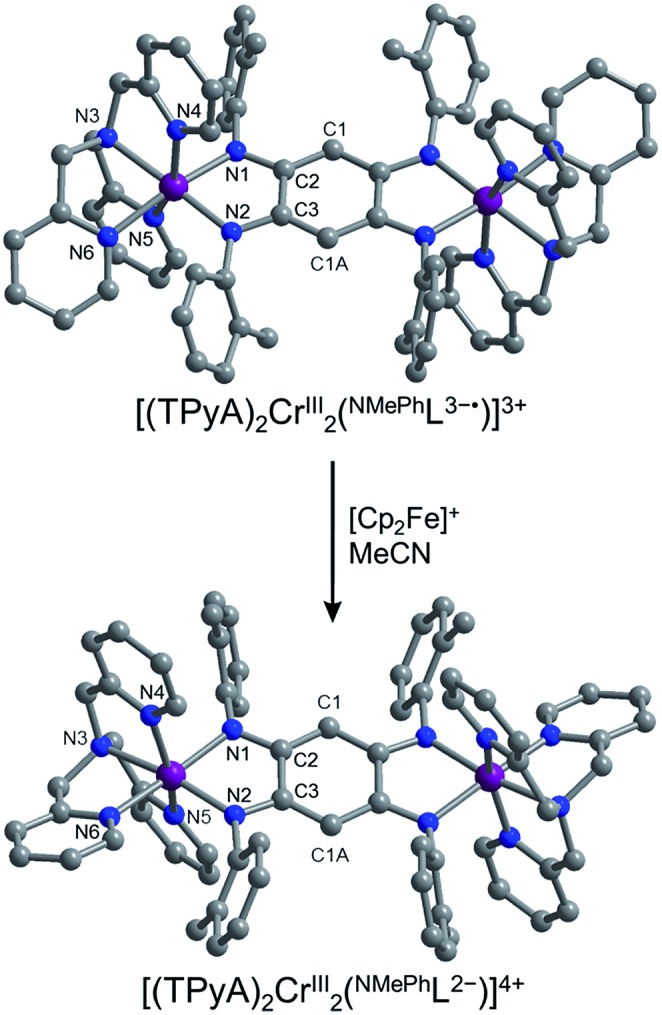
Oxidation of [(TPyA)_2_CrIII2(^NMePh^L^3–^˙)]^3+^, as observed in **5**·2.9MeCN, to give [(TPyA)_2_CrIII2(^NMePh^L^2–^)]^4+^, as observed in **1**. Purple, blue, and gray spheres represent Cr, N, and C atoms, respectively; H atoms are omitted for clarity.

Single-crystal X-ray diffraction analysis for **1**, **2**·0.4THF, **3**·2.5THF, **4**·2.5THF, and **5**·2.9MeCN was carried out at 100 K. All compounds, regardless of counteranion, crystallized in the triclinic space group *P*1[combining macron] (see Table S1†). The structures of **2**·0.4THF, **3**·2.5THF, and **4**·2.5THF feature two [BArF4]^–^ counteranions per cationic complex and are isostructural to one another, while the structure of **1** features four [BPh_4_]^–^ counteranions per cationic complex. In general, the structures of [(TPyA)_2_M_2_(^NMePh^L^2–^)]^*n*+^ consist of two crystallographically-equivalent [(TPyA)M]^*m*+^ units connected by a deprotonated ^NMePh^L^2–^ bridging ligand and related through a crystallographic site of inversion located at the center of the bridging ligand (see Fig. 1 and 2, lower). Each metal center resides in a distorted octahedral coordination environment comprising four nitrogen donor atoms from the TPyA capping ligand and two *cis*-oriented nitrogen atoms from the bridging ligand. The mean M–N distances of 2.084(7), 2.256(7), 2.203(7), and 2.165(7) Å for **1**, **2**·0.4THF, **3**·2.5THF, and **4**·2.5THF, respectively, are consistent with reported distances for high-spin Cr^III^, Mn^II^, Fe^II^, and Co^II^ ions in similar ligand environments.^19^ Within the bridging ligand for **2**·0.4THF, **3**·2.5THF, and **4**·2.5THF, the C1–C2 bond distances range from 1.402(4) to 1.408(5) Å and are slightly longer than the C3–C1A bond distances, which range from 1.375(5) to 1.383(4) Å. For **1**, this trend is reversed with the C1–C2 bond distance of 1.398(5) Å being slightly shorter than the C3–C1A distance of 1.406(4) Å. The C2–C3 bond distances for **2**·0.4THF, **3**·2.5THF, **4**·2.5THF vary from 1.495(5) to 1.499(4) Å, as expected for a typical C–C single bond, with **1** exhibiting a slightly shorter distance of 1.475(5) Å. Accordingly, the N1–C2 and the N2–C3 bond distances range from 1.312(4) to 1.337(4) Å and 1.333(5) to 1.355(4) Å, respectively, across the series. These collective distances indicate that the bridging ligand in **1**, **2**·0.4THF, **3**·2.5THF, **4**·2.5THF is best described as the dianionic, 1,4-diamido-2,5-diimino isomer ^NMePh^L^2–^.

The crystal structure of **5**·2.9MeCN exhibits an asymmetric unit that consists of two [(TPyA)_2_Cr_2_(^NMePh^L^3–^˙)]^3+^ complexes (see Fig. 2, upper) and six [BPh_4_]^–^ counteranions with an *c*-axis that is elongated relative to that of **1–4**. The two cationic complexes exhibit mean bond distances that are identical within error, despite these complexes being unrelated by any crystallographic symmetry (see Table S2†). The overall structure of the cationic complexes in **5**·2.9MeCN is similar to that in **1**, and the average Cr–N distance of 2.08(1) Å is consistent with an *S* = 3/2 Cr^III^ center.19*a* However, close comparison of the bond distances in **1** and **5**·2.9MeCN reveals several key differences. First, in moving from **1** to **5**·2.9MeCN, the C2–C3 distance decreases slightly by 2.0%, from a mean value of 1.475(5) to 1.445(9) Å, while the average benzoquinone C–N distance increases by 2.1%, from 1.335(6) to 1.363(10) Å. These differences reflect a net increase in C2–C3 bond order and a corresponding decrease in C–N bond order, consistent with an additional electron in **5**·2.9MeCN occupying a molecular orbital of primarily ligand character. Furthermore, the mean Cr–N_L_ distance decreases by 1.9%, from 2.030(4) to 1.992(7) Å, consistent with a stronger Cr–N_L_ interaction caused by moving from dianionic ^NMePh^L^2–^ to trianionic ^NMePh^L^3–^˙. In contrast, the mean Cr–N_TPyA_ distances change only slightly, increasing 0.4% from 2.111(6) to 2.119(8) Å, which supports the localization of the additional electron in **5**·2.9MeCN on the bridging ligand. These bond distances are similar to those previously observed for a ligand-centered reduction in a chloranilate radical-bridged Co_2_ complex12a,b and an azophenine radical-bridged Fe_2_ complex.^14^ Taken together, these observations suggest a configuration for the complex in **5**·2.9MeCN of [(TPyA)_2_CrIII2(^NMePh^L^3–^˙)]^3+^, perhaps resulting from a spontaneous one-electron oxidation followed by electron-transfer from Cr^II^ to ^NMePh^L^2–^, as has been observed in tetraoxolene-bridged Cr_2_ (ref. 11b) and Co_2_ complexes.11e,12a,b,^20^ Note that **5**·2.9MeCN represents only the second structurally-characterized example of a tetraazalene radical-bridged complex.^14^ Furthermore, while numerous examples of capping-ligand radicals bound to Cr^III^ are known in the literature,11a,^21^ compound **5**·2.9MeCN provides, to the best of our knowledge, the first structurally-characterized example of any radical-bridged Cr^III^ complex.

### Cyclic voltammetry

**Fig. 3:**
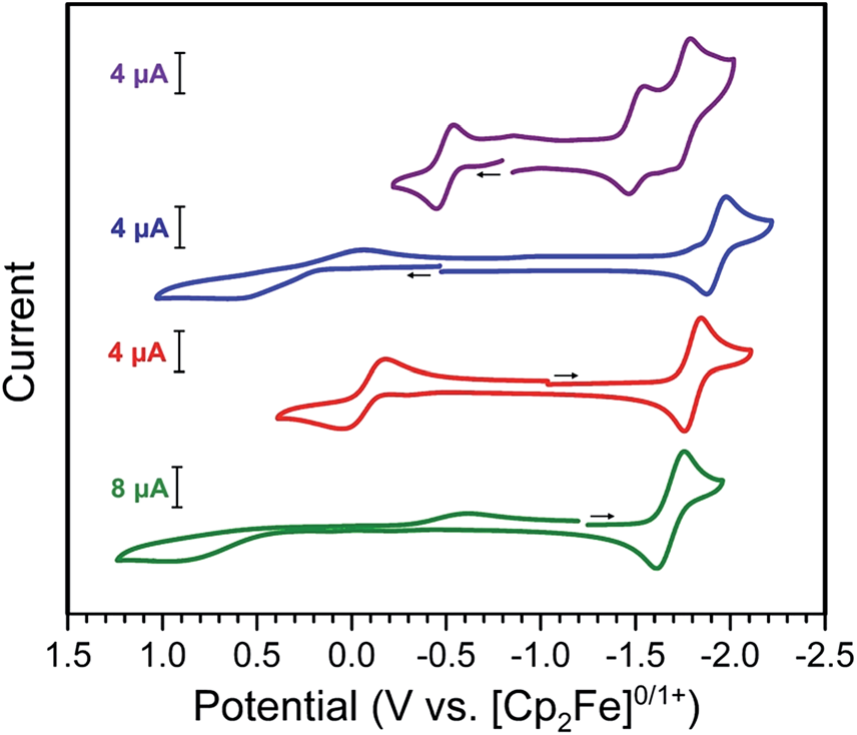
Cyclic voltammograms for solutions of **2** (Mn, blue), **3** (Fe, red), and **4** (Co, green) in THF and **5** (Cr, purple) in MeCN using a scan rate of 0.1 V s^–1^.

The cyclic voltammograms of **2–4**, as depicted in Fig. 3, each exhibit a reversible process at *E*_1/2_ = –1.93, –1.81, and –1.68 V *vs.* [Cp_2_Fe]^0/1+^ for **2**, **3**, and **4**, respectively. Considering the previously reported tetraoxolene-11b,^12^,^20^ and azophenine-bridged^14^ M_2_ complexes, in conjunction with the relative invariance of *E*_1/2_ on metal identity, we assign this event to the ligand-centered redox process ^NMePh^L^3–^˙^/2–^. The small decrease in *E*_1/2_ in moving from **2** to **3** to **4** reflects the associated increase in electronegativity of the metal center. Furthermore, the value of *E*_1/2_ = –1.81 V observed for **3** is slightly anodically shifted relative to that of –1.65 V reported for a related azophenine-bridged Fe_2_ complex,^14^ consistent with the addition of electron-donating methyl groups to the peripheral phenyl rings. The remaining redox events for **2–4** are hypothesized to be metal-based M^II/III^ couples based on their wide variation in both position and degree of reversibility.

The cyclic voltammogram of **5** exhibits three reversible processes at *E*_1/2_ = –0.50, –1.51, and –1.75 V, with an open circuit potential of –0.8 V. Based on the structural data for **5**, we assign the event centered at *E*_1/2_ = –0.50 V to the ^NMePh^L^3–^˙^/2–^ couple. In contrast, the two processes situated at more negative potentials likely correspond to metal-based Cr^II/III^ couples. Clearly, the ligand-based event at *E*_1/2_ = –0.50 V observed for **5** is positioned well positive of the ligand-centered potentials observed for **2–4**. This difference may stem from a metal-assisted mechanism where electron transfer from one chromium center stabilizes the bridging ligand in the 3–• oxidation state.11e,^20^,21c

The reversible redox event at *ca. E*_1/2_ = –1.8 V for **2–4**, in conjunction with the crystallographic evidence for a ^NMePh^L^3–^˙ ligand radical in **5**, suggests that [(TPyA)_2_M_2_(^NMePh^L^3–^˙)]^+^ (M = Mn, Fe, Co) should be chemically accessible. Toward that end, THF solutions of **2**, **3**, and **4** were treated with stoichiometric equivalents of the strong reductant (Cp)Fe(C_6_Me_6_)^22^ at –78 °C to give dark red, red-purple, and purple solutions, respectively. This immediate color change upon chemical reduction was also evident in UV/Vis/NIR spectra, which exhibited significantly different absorption profiles upon reduction that decayed back to those of the unreduced complexes at ambient temperature (see Fig. S1–S4 in the ESI†). Subsequent addition of cold hexanes to THF solutions of the reduction products at –78 °C afforded the compounds [(TPyA)_2_M_2_(^NMePh^L^3–^˙)](BArF4)·*x*[(Cp)Fe(C_6_Me_6_)](BArF4)·*y*THF (M = Mn: *x* = 1.04, *y* = 0.37 (**6**), M = Fe: *x* = 1.06, *y* = 1.6 (**7**), M = Co: *x* = 0.94, *y* = 1.2 (**8**)) as fine, microcrystalline powders that were isolated by filtration (see ESI†). Although solid samples of **6–8** are stable at –35 °C under dinitrogen atmosphere for days, solutions of these compounds undergo rapid decomposition upon exposure to air or upon standing at ambient temperature under inert atmosphere, and this instability has thus far precluded their structural characterization.

### Mössbauer spectroscopy

**Fig. 4:**
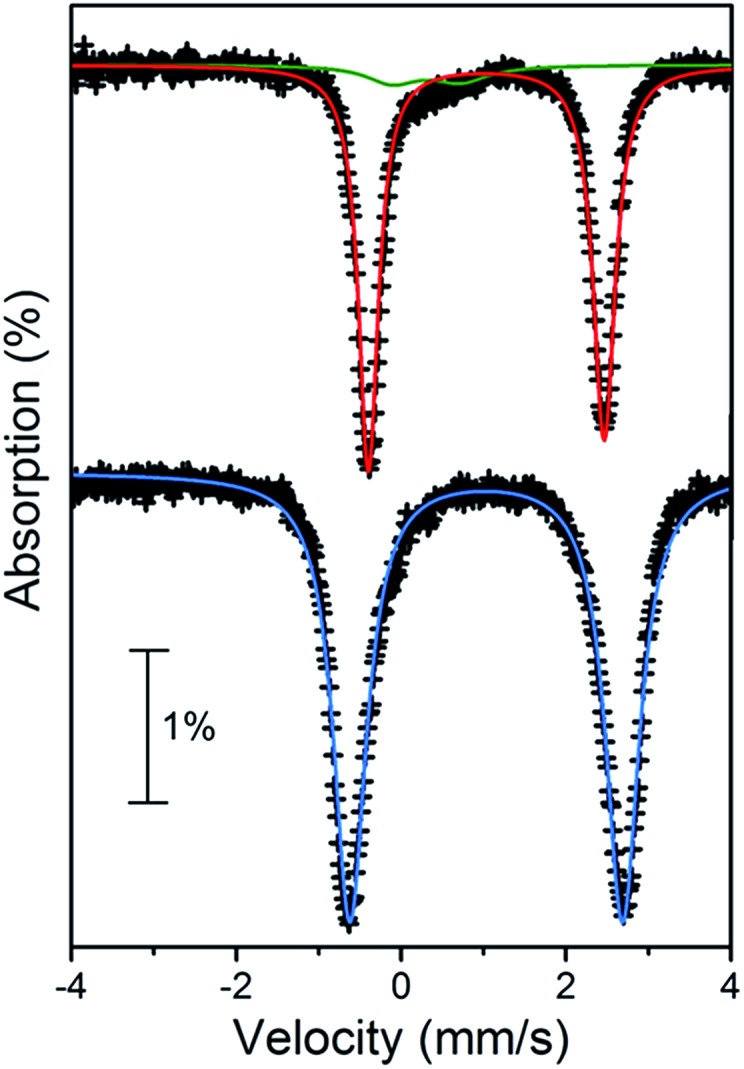
Mössbauer spectra for **3** (upper) and **7′** (lower) taken at 80 K. Red and blue lines correspond to fits to high-spin Fe^II^ while the green line indicates a small amount of Fe^III^-containing impurity.

To confirm the presence of a ligand-centered process upon reduction of **3**, zero-field Mössbauer spectra were collected for solid samples of **3** and its reduction product. In order to avoid convolution of the spectra, the chemical reductant (C_5_Me_5_)_2_Co was employed as the chemical reductant in place of (Cp)Fe(C_6_Me_6_). Here, a THF solution containing **3** was treated with (C_5_Me_5_)_2_Co at –78 °C. Subsequent addition of cold hexanes into this reaction mixture yielded the compound [(TPyA)_2_Fe_2_(^NMePh^L^3–^˙)](BArF4)·1.35[(C_5_Me_5_)_2_Co](BArF4) (**7′**) as a dark red microcrystalline powder. At 80 K, the Mössbauer spectrum of **3** exhibits a major quadrupole doublet and a second minor doublet that we assign to a small amount of Fe^III^-containing impurity (see Fig. 4, upper). A fit to the major doublet gives an isomer shift of *δ* = 1.026(4) mm s^–1^ and a quadrupole splitting of Δ*E*_Q_ = 2.856(3) mm s^–1^, which are in good agreement with reported high-spin Fe^II^ centers in similar coordination environments.^14^,^23^,^24^ Likewise, the spectrum obtained for **7′** exhibits a symmetric quadrupole doublet that can be fit to give parameters of *δ* = 1.032(1) mm s^–1^ and a slightly larger quadrupole splitting of Δ*E*_Q_ = 3.307(4) mm s^–1^ (see Fig. 4, lower). The nearly identical isomer shifts in **3** and **7′** provides strong support for a ligand-based reduction in moving from **3** and **7′**, as was previously observed in a related azophenine-bridged Fe_2_ complex.^14^ This observation, in conjunction with the cyclic voltammetric data across the series and magnetic behavior (see below), supports the assignment of the cationic complexes in **6–8** as radical-bridged [(TPyA)_2_MII2(^NMePh^L^3–^˙)]^+^. Finally, the slight increase in quadrupole splitting of **7′** compared to **3** likely stems from the change in ligand field upon reduction and possible distortion from an octahedral coordination environment at Fe, as was observed in the azophenine-bridged analogue.^14^

### Static magnetic properties

**Fig. 5:**
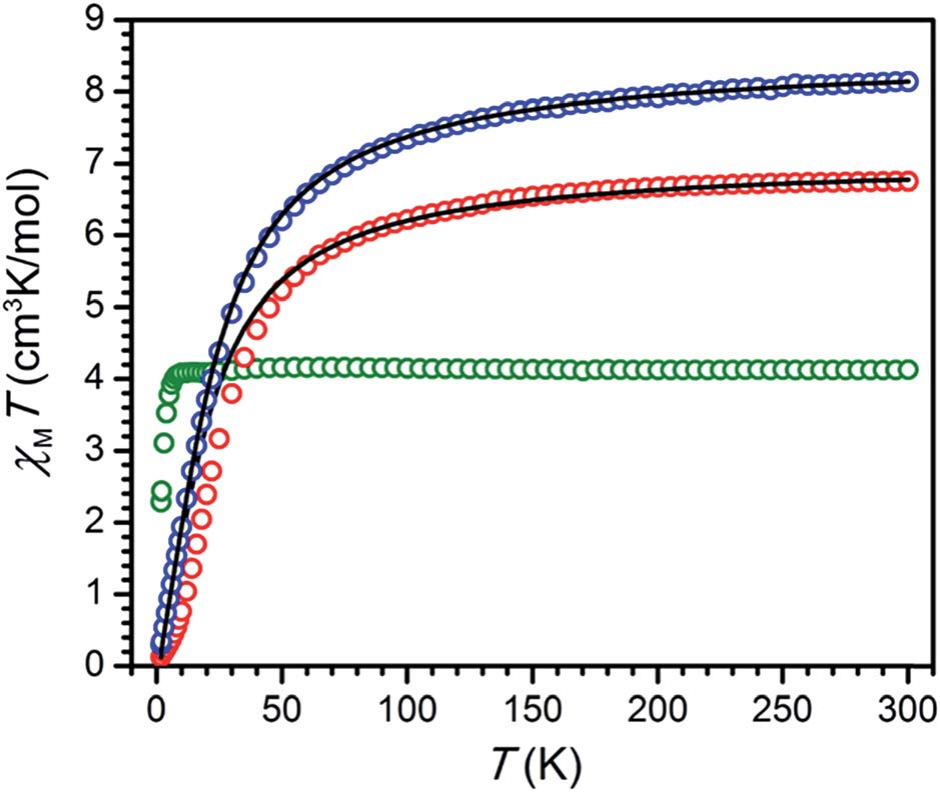
Variable-temperature dc magnetic susceptibility for **2** (Mn, blue), **3** (Fe, red), and **4** (Co, green) collected under an applied field of 1 T. Black lines indicate fits to data.

To probe and compare magnetic exchange interactions in **2–8**, variable-temperature dc magnetic susceptibility measurements were carried out on solid samples under applied dc fields of 1 or 2 T. Measurements for **2–5** were carried out in the temperature range 1.8–300 K, while data for **6–8** were collected up to only 270 K in order to prevent thermal decomposition. The resulting plots of *χ*_M_*T vs. T* for **2–4** are shown in Fig. 5. At 300 K, the compounds exhibit values of *χ*_M_*T* = 8.13, 6.75, and 4.13 cm^3^ K mol^–1^ for **2**, **3**, and **4**, respectively, corresponding to two magnetically non-interacting *S* = 5/2, 2, and 3/2 metal centers. As the temperature is decreased, the data, with the exception of **4**, undergo a gradual then rapid decline, reaching minimum values of 0.30 and 0.13 cm^3^ K mol^–1^ at 1.8 K for **2** and **3**, respectively, corresponding to population of *S* = 0 ground states. This decrease in *χ*_M_*T* with lowering temperature is indicative of weak antiferromagnetic coupling between metal centers *via* a superexchange mechanism through the diamagnetic ^NMePh^L^2–^ bridging ligand. To quantify this interaction, the data were fit to the Van Vleck equation according to the spin Hamiltonian *Ĥ* = –2*J*(*ŝ*_M1_·*ŝ*_M2_) to give exchange constants of *J* = –1.64(1) and –2.16(2) cm^–1^ and *g* = 1.97 and 2.18 for **2** and **3**, respectively (see Table 1).

**Table 1:** Summary of parameters obtained from fits and simulations of magnetic data

		[(TPyA)_2_M_2_(L^2–^)]^*n*+^	[(TPyA)_2_M_2_(L^3–^˙)]^(*n*–1)+^
M = Cr^III^	*J* (cm^–1^)	—	–626(7)
	*D* ^*a*^ (cm^–1^)	—	+0.6
M = Mn^II^	*J* (cm^–1^)	–1.64(1)	–157(7)
	*D* (cm^–1^)	—	+0.4
M = Fe^II^	*J* (cm^–1^)	–2.16(2)	–307(9)
	*D* (cm^–1^)	—	–13.8
M = Co^II^	*J* (cm^–1^)	>0^*b*^	–396(16)
	*D* (cm^–1^)	—	–10.7

^*a*^These values of *D* were obtained from fitting reduced magnetization data.

^*b*^No fit was obtained for these data.

In contrast, the temperature dependence of *χ*_M_*T* observed for **4** is not consistent with population of an *S* = 0 ground state at low temperature. Rather, the slight decline in *χ*_M_*T* with lowering temperature between 65 and 20 K likely stems from large spin–orbit coupling, as expected for high-spin Co^II^ centers.^25^ As temperature is further decreased from 20 K, the *χ*_M_*T* data reach a plateau and then undergo a precipitous decrease to a minimum value of *χ*_M_*T* = 2.29 cm^3^ K mol^–1^ at 1.8 K, indicative of a weak ferromagnetic superexchange interaction between Co^II^ centers *via* the ^NMePh^L^2–^ bridging ligand together with intermolecular antiferromagnetic interactions and/or zero-field splitting (see Fig. S5†). Such competition of different parameters in the overall temperature regime precludes a reliable estimation of the magnetic exchange constant for **4** based on a simple isotropic exchange spin Hamiltonian.

**Fig. 6:**
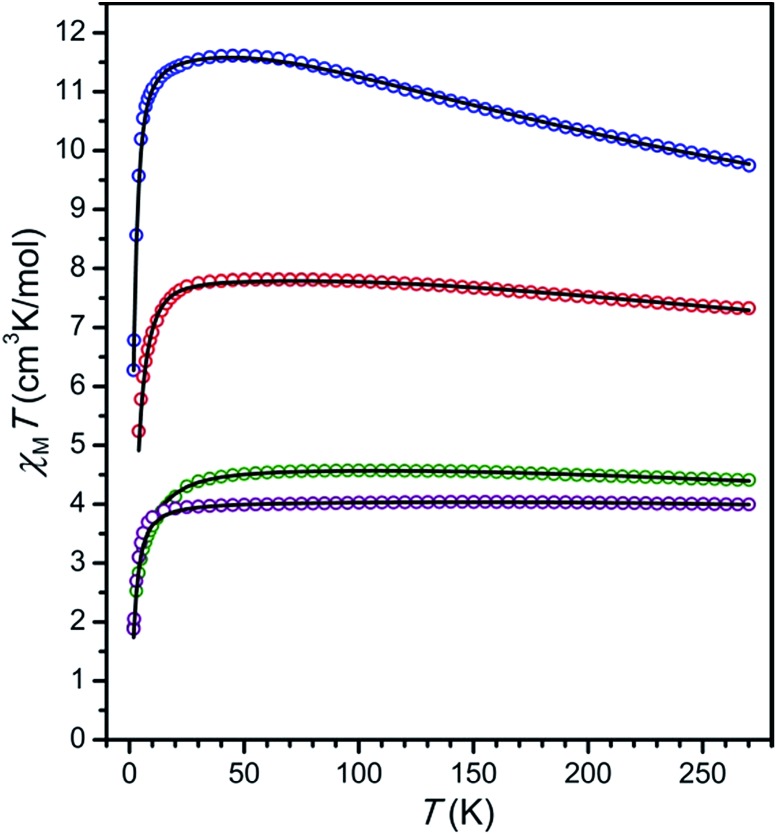
Variable-temperature dc magnetic susceptibility for **5** (Cr, purple), **6** (Mn, blue), **7** (Fe, red), and **8** (Co, green) and under an applied field of 1 (**6–8**) or 2 (**5**) T. Black lines represent simulations to the data.

The plots of *χ*_M_*T vs. T* for compounds **5–8** exhibit a markedly different profile than those for compounds **1–4** (see Fig. 6). At 270 K, the data provide values of *χ*_M_*T* = 4.00, 9.75, 7.33, and 4.41 cm^3^ K mol^–1^ for **5**, **6**, **7**, and **8**, respectively. As temperature is decreased, the data undergo a gradual increase and reach maximum values of *χ*_M_*T* = 4.03, 11.61, 7.81, and 4.57 cm^3^ K mol^–1^ at 150, 45, 55, and 95 K for **5**, **6**, **7**, and **8**, respectively, albeit with slightly different profile shapes. This temperature dependence suggests the presence of two metal centers strongly antiferromagnetically coupled to an *S* = 1/2 radical, giving rise to *S* = 5/2, 9/2, 7/2, and 5/2 ground states for **5–8**, respectively. While the relative lack of curvature in the plots of *χ*_M_*T vs. T* precludes an exact determination of coupling strength in these compounds, these values can nevertheless be estimated through simulations to the data of **5–8** according to the spin Hamiltonian *Ĥ* = –2*J*[*ŝ*_rad_·(*ŝ*_M1_ + *ŝ*_M2_)].^26^ These simulations, shown as black lines in Fig. 6, provide estimated exchange constants of *J* = –626(7), –157(7), –307(9), and –396(16) cm^–1^ for **5**, **6**, **7**, and **8**, respectively (see Table 1) and *g* = 1.96, 1.95, 2.01 and 2.07, respectively.

The precipitous decline below 40 K can be attributed to a variety of effects, most commonly zero-field splitting and antiferromagnetic inter- or intra-molecular M···M interactions. In order to avoid over-parameterization, only one parameter, either an axial zero-field splitting parameter of a metal ion (*D*_M_) or an intermolecular magnetic exchange constant (*zJ*′), was included in the simulation of each dataset. Simulations to data with the incorporation of a *D*_M_ term in the Hamiltonian^27^ give axial zero-field splitting parameter estimates of *D*_M_ = –5.9 and –15.5 cm^–1^, for **7** and **8**, respectively. Given the negligible magnetic anisotropy of the high-spin, octahedral Cr^III^ and Mn^II^ ions, an intermolecular magnetic coupling term using the mean-field approximation, rather than a zero-field splitting term, was included in the simulations of **5** and **6**.^28^ These simulations give values of the intermolecular exchange constant of *zJ*′ = –1.35 and –0.33 cm^–1^, respectively. Note that the estimated values of *D*_M_ or *zJ*′ are likely high due to contribution of other effects at low temperature. Importantly, note that the introduction of zero-field splitting or intermolecular exchange terms in the simulation does not significantly affect the magnitude of intramolecular metal–radical coupling.

**Fig. 7:**
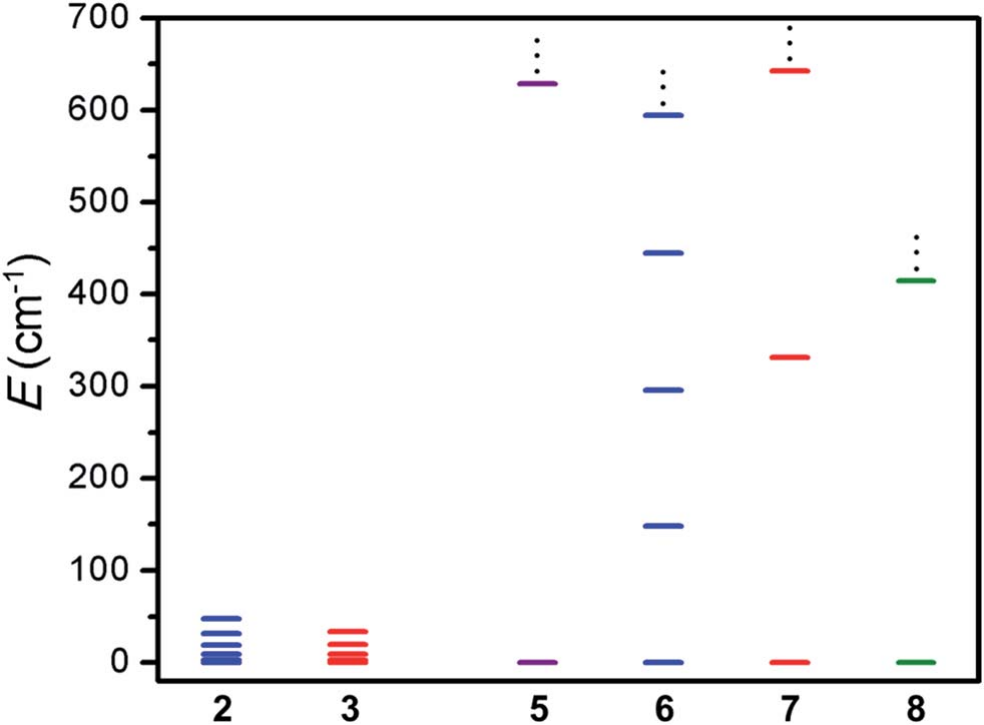
Spin ladder showing the lowest spin energy levels for compounds **2**, **3**, and **5–8**, as calculated from fits or simulations of the magnetic susceptibility data. Purple, blue, red, and green lines correspond to M = Cr, Mn, Fe, and Co, respectively.

Remarkably, the values of *J* obtained for **6** and **7** represent 95- and 142-fold enhancement of magnetic exchange strength compared to those obtained for **2** and **3**, owing to the direct overlap of magnetic orbitals between each metal center and the paramagnetic bridging ligand. This dramatic increase in exchange strength serves to isolate the spin ground state in the radical-bridged complexes to the extent that the lowest lying spin excited state is situated well above the entire spin manifold of the ^NMePh^L^2–^-bridged analogues (see Fig. 7). Such a large increase of exchange strength upon undergoing oxidation or reduction is rare, with similar magnitudes of enhancement in complexes limited to tetraoxolene radical-bridged CoII2 (ref. 12*a* and *b*) and CrIII2 (ref. 12*c*) complexes, a nindigo radical-bridged CoII2 complex,^29^ and an azophenine radical-bridged FeII2 complex.^14^

Moreover, to our knowledge, **5** exhibits the strongest magnetic exchange yet reported between Cr^III^ and a ligand radical in a multinuclear system, although an interaction of a qualitatively similar strength has been observed in a chloranilate radical-bridged CrIII2 complex.12*c* The value of *J* obtained for **6** is similar in magnitude to the largest value previously reported for an octahedral Mn^II^ complex of any nuclearity, with a slightly larger coupling constant of *J* = –172 cm^–1^ observed in a Mn^II^ chain bridged by a nitronyl nitroxide radical ligand.^30^ The value of *J* found for **7** represents the second strongest magnetic exchange yet reported in a multinuclear Fe^II^ complex, eclipsed only by a related azophenine radical-bridged Fe_2_ complex.^14^ This lower value likely stems from the increased steric bulk in **7** induced by the addition of a methyl group onto the peripheral phenyl rings of the bridging ligand. Finally, to the best of our knowledge, **8** represents the strongest metal–radical coupling yet reported in a multinuclear cobalt complex, surpassing the previous record of *J* = –133 cm^–1^ held by a nindigo-radical bridged CoII2 complex.^29^ Taken together, this series of molecules demonstrates the ability of tetraazalene radicals to mediate very strong coupling across a wide range of transition metal complexes.

**Fig. 8:**
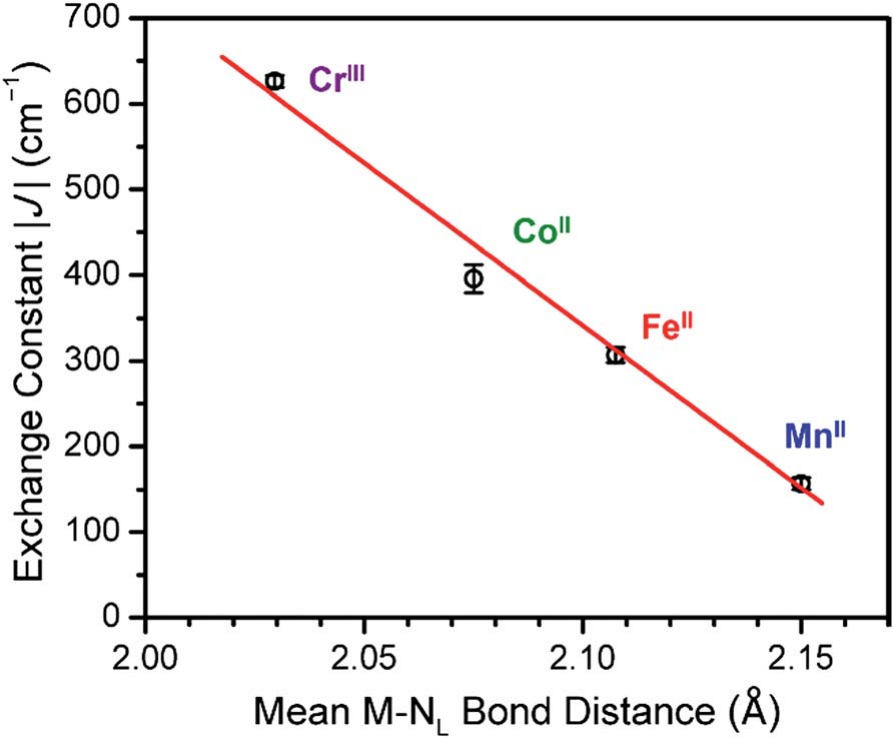
Linear relationship between mean M–N_L_ bond distance for **1–4** and the obtained magnitude of metal–ligand radical exchange constant |*J*| in **5–8**.

While dc magnetic susceptibility data for **5–8** indicate very strong antiferromagnetic metal–ligand radical interaction for all complexes, the magnitude of this interaction varies widely, from *J* = –157(7) cm^–1^ for M = Mn^II^ to *J* = –626(7) cm^–1^ for M = Cr^III^. Although the lack of structural characterization of **6–8** precludes the possibility of a direct magnetostructural correlation in these complexes, careful examination of the bond distances in the oxidized analogues **1–4** reveals a strong linear correlation between the mean M–N_L_ bond distance in **1–4** and *J* in the corresponding radical-bridged **5–8** (see Fig. 8). Here, we assume that while M–N_L_ distances in the ^NMePh^L^2–^- and ^NMePh^L^3–^˙-bridged complexes will differ, the trend of M–N_L_ distance as a function of metal will be similar in the two series. This distance increases by a total of 5.9% in moving from **1** (Cr^III^) to **4** (Co^II^) to **3** (Fe^II^) to **2** (Mn^II^), in line with decreasing effective nuclear charge, and this change is associated with a corresponding decrease in *J* of 75% upon moving from **5** (Cr^III^) to **8** (Co^II^) to **7** (Fe^II^) to **6** (Mn^II^). This correlation suggests that the strength of coupling in this series depends primarily on effective nuclear charge rather than changes in electronic population of d orbitals. Note that a correlation between M–L bond distance and superexchange strength has been previously probed both experimentally and theoretically in a number of molecular complexes and extended solids, which revealed an exponential decay of the coupling strength with increasing M–L distance.^31^ Moreover, a theoretical study on a mononuclear Cr^III^–semiquinone complex demonstrated that this exponential dependence can also be observed in the case of direct exchange.^32^

To probe the magnetic anisotropy and confirm the spin ground states of compounds **5–8**, low-temperature magnetization data were collected at selected dc fields (see Fig. S6–S9†). For compounds **5** and **6**, the isofield curves are nearly superimposed and reveal a saturation of the magnetization under an applied field of 7 T that falls close to the values of *M* = 5 and 9 μ_B_ expected for *S* = 5/2 and 9/2 ground states (*g* = 2), respectively. This behavior indicates the presence of relatively weak magnetic anisotropy, as is expected for octahedral Cr^III^ and high-spin Mn^II^ metal centers. Magnetization data for **7** and **8**, however, exhibit large splitting of the isofield curves and saturate well below the values *M* = 7 and 5 μ_B_ expected for *S* = 7/2 and 5/2 ground states (*g* = 2), respectively, highlighting the presence of strong magnetic anisotropy. To quantify the anisotropy across the series, the reduced magnetization data were fit to give parameters of *D* = +0.6, +0.4, –13.8, and –10.7 cm^–1^ and *g* = 1.83, 1.88, 2.13, and 2.02 for **5–8**, respectively.^33^ Note that the value of *D* = –13.8 cm^–1^ obtained for **7** is, to our knowledge, the largest yet observed in a multinuclear single-molecule magnet (see below), surpassing the value of –8.4 cm^–1^ previously reported for a related azophenine-radical bridged Fe_2_ complex.^14^

### Dynamic magnetic properties

**Fig. 9:**
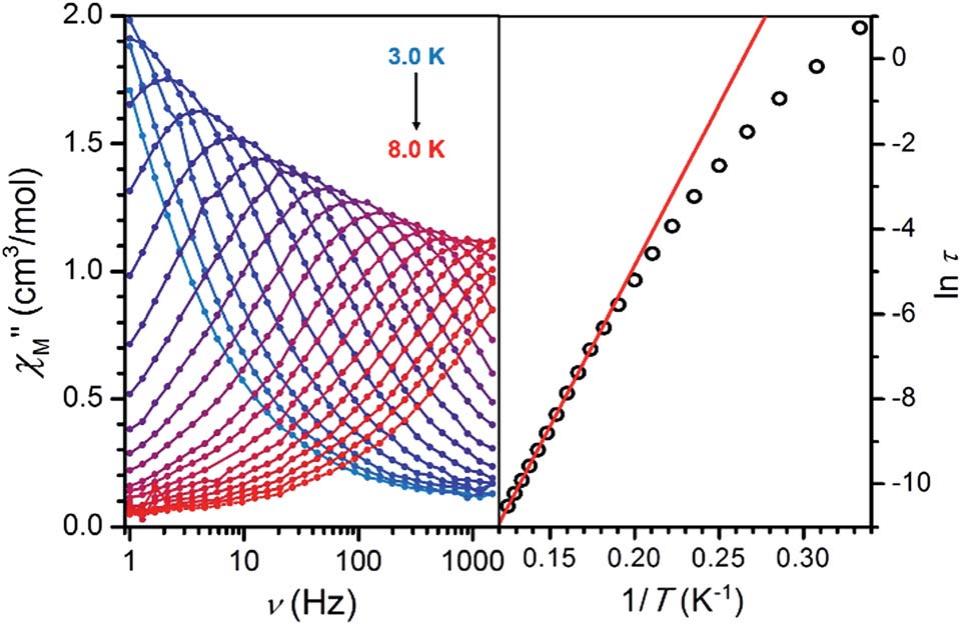
Left: Variable-frequency out-of-phase ac susceptibility data for **7**. Right: Arrhenius plot of relaxation time, with a fit to linear region giving *U*_eff_ = 52(1) cm^–1^.

Finally, variable-frequency ac susceptibility data under zero applied dc field were collected in order to probe single-molecule magnet behavior for each compound with an *S* > 0 spin ground state. Despite the presence of considerable magnetic anisotropy in several compounds (see Table 1), only **7** exhibits a frequency-dependent peak in the out-of-phase component (*χ*′′_M_) of the ac susceptibility (see Fig. 9, left). The corresponding Arrhenius plot of relaxation time for **7** (see Fig. 9, right) shows a linear region at higher temperatures between 5.75 and 8.0 K, indicative of a single-molecule magnet. A fit to the data in this temperature range gives a spin relaxation barrier *U*_eff_ = 52(1) cm^–1^ with *τ*_0_ = 2.1(1) × 10^–9^ s. These values are close to those of *U*_eff_ = 50(1) cm^–1^ and *τ*_0_ = 2.7(2) × 10^–10^ s previously reported for a related azophenine radical-bridged Fe_2_ complex.^14^

## Conclusions

The foregoing results demonstrate the ability of tetraazalene radicals to promote exceptionally strong coupling across a range of transition metal complexes. Specifically, the radical-bridged complexes [(TPyA)_2_M_2_(^NMePh^L^3–^˙)]^*n*+^ (*n* = 3: M = Cr^III^, *n* = 1: Mn^II^, Fe^II^, Co^II^) were synthesized and shown to exhibit exchange constants of *J* = 626(7), 157(7), 307(9), and 396(16) cm^–1^ for M = Cr^III^, Mn^II^, Fe^II^, and Co^II^, respectively, owing to direct exchange between metals and ligand radical. The large variation in the strength of the magnetic coupling in the radical-bridged complexes shows a strong correlation with the mean metal to bridging ligand bond distance in the ^NMePh^L^2–^ analogue of each, revealing almost linear enhancement of the magnetic coupling with decreasing bond distance. Work is underway to incorporate tetraazalene radical ligands into higher-dimensional magnetic solids.

## Supplementary Material

Supplementary information

Crystal structure data
