# Supplementary material for: A series of tetraazalene radical-bridged M2 (M = CrIII, MnII, FeII, CoII) complexes with strong magnetic exchange coupling
Source: Chem Sci. 2015 Aug 18;6(11):6639–48. doi: 10.1039/c5sc02725j (PMC5802272; doi:10.1039/c5sc02725j)
Supplement: Supplementary file 1 [file SC-006-C5SC02725J-s001.pdf]

Electronic Supplementary Material (ESI) for Chemical Science.
This journal is © The Royal Society of Chemistry 2015

Electronic Supplementary Information for:

A Series of Tetraazalene Radical-Bridged M_2 ($M = Cr^{III}, Mn^{II}, Fe^{II}, Co^{II}$) Complexes with Strong Magnetic Exchange Coupling

Jordan A. DeGayner, Ie-Rang Jeon, T. David Harris*

Department of Chemistry, Northwestern University, 2145 Sheridan Road, Evanston IL 60208-3113
email: dharris@northwestern.edu

Chemical Science

Table of Contents

Experimental Section **S2**
Table S1: Crystallographic data for compounds **S7**
Table S2: Selected interatomic distances and angles for compounds **S8**
Figure S1: UV/visible/NIR for **5** **S9**
Figure S2: UV/visible/NIR for **2** and **6** **S10**
Figure S3: UV/visible/NIR for **3** and **7** **S11**
Figure S4: UV/visible/NIR for **4** and **8** **S12**
Figure S5: Variable-temperature dc magnetic susceptibility data for **4** **S13**
Figure S6: Low-temperature variable-field magnetization data for **5** **S14**
Figure S7: Low-temperature variable-field magnetization data for **6** **S15**
Figure S8: Low-temperature variable-field magnetization data for **7** **S16**
Figure S9: Low-temperature variable-field magnetization data for **8** **S17**
Figure S10: 100 K Variable-field data for **2** **S18**
Figure S11: 100 K Variable-field data for **3** **S19**
Figure S12: 100 K Variable-field data for **4** **S20**
Figure S13: 100 K Variable-field data for **5** **S21**
Figure S14: 100 K Variable-field data for **6** **S22**
Figure S15: 100 K Variable-field data for **7** **S23**
Figure S16: 100 K Variable-field data for **8** **S24**
Figure S17: In-phase ac magnetic susceptibility for **7** **S25**
References **S26**

Experimental Section

General Considerations. The manipulations described below were performed under a dinitrogen atmosphere in a Vacuum Atmospheres Nexus II glovebox unless otherwise noted. Glassware was oven-dried at 150 °C for at least 4 h and allowed to cool in an evacuated antechamber prior to use in the glovebox. Acetonitrile (MeCN), diethyl ether (Et_2O), hexanes, tetrahydrofuran (THF), and toluene were dried using a commercial solvent purification system from Pure Process Technology and stored over 3 or 4 Å molecular sieves prior to use. Deuterated solvents were purchased from Cambridge Isotope Labs, deoxygenated by three successive freeze-pump-thaw cycles, and stored over 3 or 4 Å molecular sieves prior to use. The reagent *o*-toluidine was deoxygenated by three successive freeze-pump-thaw cycles and stored over 4 Å molecular sieves prior to use. Diethyl ether, hexanes, tetrahydrofuran and toluene were typically tested with a standard purple solution of sodium benzophenone ketyl in THF in order to confirm effective oxygen and moisture removal. The compounds Ag(BAr^F_4) (BAr^F_4 = tetrakis[3,5-bis(trifluoromethyl)phenyl]borate), [M(MeCN)$_6$](BAr^F_4)$_2$ (M = Mn, Fe, Co),[1] tris(2-pyridylmethyl)amine (TPyA),[2] lithium bis(trimethylsilyl)amide (Li[N(SiMe$_3$)$_2$]),[3] Tl(BAr^F_4),[4] and ferrocenium tetraphenylborate ([Cp$_2$Fe](BPh$_4$))[5] were prepared as previously reported. All other reagents were purchased from commercial vendors and used without further purification.

[(Cp)Fe(C$_6$Me$_6$)]PF$_6$. This compound was prepared according to a modified literature procedure.[6] Hexamethylbenzene (1.09 g, 6.73 mmol) was added to freshly ground Al metal (0.0830 g, 3.10 mmol), $AlCl_3$ (1.80 g, 13.5 mmol), and ferrocene (1.25 g, 6.73 mmol) in 20 mL methylcyclohexane, and the resulting mixture was heated at reflux for 12 h. The resulting dark mixture was then carefully quenched with H_2O (50 mL), and the orange organic layer was removed. The remaining aqueous green solution was washed with petroleum ether (3 × 10 mL). To the aqueous solution was then added (NH_4)PF_6 (1.30 g, 8.10 mmol) in H_2O (10 mL), and the resulting suspension was filtered to give a dark yellow powder. This powder was washed with an excess of H_2O (30 mL), dissolved in acetone (15 mL), and then was precipitated with Et_2O (50 mL). The solid was collected on a nylon membrane filter (0.22 μm) and washed with successive aliquots of Et_2O (3 × 5 mL) to afford the product as a bright yellow solid (0.577 g, 20%). ^{1}H NMR (CD$_3$CN): 4.52 (s, 5H), 2.45 (s, 18H).

(Cp)Fe(C$_6$Me$_6$). This compound was prepared according to a modified literature procedure.[7] The compound [(Cp)Fe(C$_6$Me$_6$)]PF$_6$ (1.00 g, 2.34 mmol) was dissolved in THF (15 mL), and the resulting yellow solution was added to finely-ground NaHg amalgam (4% Na wt/wt, 6 g) to afford a dark green solution. After stirring for 6 h, the solution was dried under reduced pressure. The ensuing dark green residue was dissolved in hexanes (15 mL), and the solution was filtered through diatomaceous earth. The filtrate was then dried under reduced pressure to give the product as a green solid (0.588 g, 89%). ^{1}H NMR (C$_6$D$_6$): −7.07 (s, 18H), −40.31 (s, 5H).

[Cr(MeCN)$_6$](BAr^F_4)$_2$. This compound was prepared according to a modified literature procedure.[1,4] A solution of $CrCl_2$ (63.3 mg, 0.293 mmol) in MeCN (6 mL) was added to a solution of Tl(BAr^F_4) (626 mg, 0.586 mmol) in MeCN (10 mL). The resulting light green suspension was stirred at ambient temperature for 5 h and then filtered through diatomaceous earth to remove a white insoluble solid. The pale green filtrate was concentrated under reduced pressure to ca. 1 mL and stored at −35 °C for 12 h to give blue needle-shaped crystals. The crystals were collected by filtration and washed with cold (−45 °C) MeCN to give 145 mg (54%) of product. FT-IR (ATR, cm^{-1}): 2327 (w), 2299 (w), 1611 (m), 1358 (s), 1285 (s), 1181 (m, sh), 1128 (b, s), 889 (m), 838 (m), 711, (m), 683 (m), 671 (m).

***N, N′, N″, N‴* -tetra(2-methylphenyl)-2,5-diamino-1,4-diiminobenzoquinone ($^{NMePh}LH_2$).** This compound was prepared according to a modified literature procedure.[8] A Pd catalyst was prepared by combining 1,3-bis(2,6-diisopropylphenyl)imidazolium choride (0.128 g, 0.300 mmol), NaO*t*Bu (0.0450 g, 0.468 mmol), $Pd(OAc)_2$ (0.0375 g, 0.165 mmol), and toluene (5 mL) in a 20 mL vial, resulting in an orange mixture. The mixture was stirred at 80 °C for 10 minutes, and then it was added to a 500 mL reaction flask containing 1,2,4,5-tetrabromobenzene (2.22 g, 5.64 mmol) suspended in toluene (100 mL). The reagents *o*-toluidine (3.11 g, 29.0 mmol) and NaO^tBu (2.65 g, 27.6 mmol) were then added to the flask, and the resulting light orange mixture was stirred at 110 °C for 12 h to give a dark mixture. After being allowed to cool to 40 °C, toluene was removed from the reaction mixture under reduced pressure, and the ensuing dark solid was washed with hexanes (150 mL) until the rinses were colorless. The solid was further washed with MeOH (50 mL), and the residue was extracted into CH_2Cl_2 (100 mL). The resulting dark blue filtrate was dried under reduced pressure and then dissolved in $CHCl_3$ (200 mL). Dioxygen gas was bubbled into this solution for 18 h, resulting in a dark orange solution. Removal of solvent under reduced pressure gave $^{NMePh}LH_2$ as a bright orange powder (2.15 g, 79%). ^1H NMR ($CDCl_3$): 8.22 (s, 2H), 7.10-7.22 (br m, 8H), 6.92-7.04 (br m, 8H), 5.94 (s, 2H), 2.21 (s, 12H). IR (ATR, cm^{-1}): ν(N-H) 3282.

Crystallization of $[(TPyA)_2Cr_2(^{NMePh}L^{2-})](BPh)_4·4MeCN$ (1). Solid $[Cp_2Fe](BPh_4)$ (16.5 mg, 0.0327 mmol) in MeCN (5 mL) was added dropwise with stirring to a solution of **5** (69.0 mg, 0.0311 mmol) in MeCN (3 mL). The resulting dark brown solution was stirred at ambient temperature for 1 h. Vapor diffusion of Et_2O (20 mL) into this solution yielded a mixture a compounds that included **1** as brown plate-shaped crystals suitable for single-crystal X-ray diffraction. This compound could not be isolated in pure bulk form (see Results and Discussion).

$[(TPyA)_2Mn_2(^{NMePh}L^{2-})](BAr^F_4)_2$ (2). TPyA (0.0283 g, 0.0975 mmol) was dissolved in THF (3 mL), and this solution was added dropwise with stirring to a solution of $[Mn(MeCN)_6](BAr^F_4)_2$ (0.197 g, 0.0972 mmol) in THF (3 mL). The resulting golden solution was stirred for 5 min at ambient temperature and then was treated with solid $^{NMePh}LH_2$ (0.0241 g, 0.0485 mmol). To this mixture was added dropwise with stirring a solution of $Li[N(SiMe_3)_2]$ (0.0185 g, 0.110 mmol) in THF (5 mL), resulting in a dark brown solution. After stirring at ambient temperature for 12 h, the brown solution was filtered through diatomaceous earth, and careful layering of hexanes onto the filtrate afforded **2**·0.4THF as dark yellow needle-shaped crystals suitable for single-crystal X-ray diffraction. Subsequent washing of the crystals with cold (−78 °C) EtOH followed by hexanes removed co-precipitated $Li(BAr^F_4)$. The remaining crystals were dried under reduced pressure at ambient temperature for 12 h in order to remove the solvent molecules, yielding **2** (0.0975 g, 69%) as a dark yellow crystalline solid. Anal. Calcd. for $C_{134}H_{90}F_{48}Mn_2N_{12}B_2$: C, 55.3; H, 3.12; N, 5.77%. Found: C, 54.7; H, 3.19; N, 5.39%. Absorption spectrum (THF*): λ_{max}* = 428 nm.

$[(TPyA)_2Fe_2(^{NMePh}L^{2-})](BAr^F_4)_2$ (3). TPyA (0.0523 g, 0.180 mmol) was dissolved in THF (3 mL), and this solution was added dropwise to a solution of $[Fe(MeCN)_6](BAr^F_4)_2$ (0.366 g, 0.180 mmol) in THF (3 mL). The resulting light red solution was stirred for 5 min at ambient temperature and then was treated with solid $^{NMePh}LH_2$ (0.0447 g, 0.090 mmol). To this mixture was added dropwise with stirring a solution of $Li[N(SiMe_3)_2]$ (0.0320 g, 0.191 mmol) in THF (5 mL), resulting in a dark brown solution. After stirring at ambient temperature for 12 h, the brown solution was filtered through diatomaceous earth, and careful layering of hexanes onto the filtrate afforded **3**·2.5THF as dark brown needle-shaped crystals suitable for single-crystal X-ray diffraction. Subsequent washing of the crystals with cold (−78 °C) ethanol followed by hexanes enabled removal of co-precipitated $Li(BAr^F_4)$. The obtained crystals

were dried under reduced pressure at ambient temperature for 12 h in order to remove the solvent molecules, yielding **3** (0.130 g, 50%) as a dark brown crystalline solid. Anal. Calcd. for $C_{134}H_{90}F_{48}Fe_2N_{12}B_2$: C, 55.2; H, 3.11; N, 5.77%. Found: C, 54.0; H, 3.27; N, 5.47%. Absorption spectrum (THF): λ_{max} = 435 nm.

$[(TPyA)_2Co_2(^{NMePh}L^{2-})](BAr^F{}_4)_2$ (4). TPyA (0.052 g, 0.18 mmol) was dissolved in THF (5 mL), and this solution was added dropwise to a solution of $[Co(MeCN)_6](BAr^F{}_4)_2$ (0.36 g, 0.18 mmol) in THF (3 mL). The resulting golden solution was stirred for 5 min at ambient temperature and then was treated with solid $^{NMePh}LH_2$ (0.044 g, 0.089 mmol). To this mixture was added dropwise with stirring a solution of $Li[N(SiMe_3)_2]$ (0.035 g, 0.21 mmol) in THF (5 mL), resulting in a dark brown solution. After stirring at ambient temperature for 12 h, the solution was filtered through diatomaceous earth, and careful layering of hexanes onto the filtrate gave **4**·2.5THF as dark yellow, needle-shaped crystals suitable for single-crystal X-ray diffraction. The obtained crystals were dried under reduced pressure at ambient temperature for 2 h in order to remove the solvent molecules, yielding **4** (0.18 g, 70%) as a dark yellow crystalline solid. Anal. Calcd. for $C_{134}H_{90}F_{48}Co_2N_{12}B_2$ C 55.1, H 3.11, N 5.76 %. Found: C, 55.2; H, 3.23; N, 5.66 %. Absorption spectrum (THF): λ_{max} = 450 nm.

$[(TPyA)_2Cr_2(^{NMePh}L^{3-\bullet})](BPh)_3\cdot1.4MeCN$ (5). TPyA (0.0720 g, 0.248 mmol) was dissolved in THF (5 mL), and this solution was added dropwise to a solution of $[Cr(MeCN)_6](BAr^F{}_4)_2$ (0.503 g, 0.248 mmol) in THF (3 mL). The resulting brown solution was stirred for 5 min at ambient temperature and then was treated with solid $^{NMePh}LH_2$ (0.0616 g, 0.124 mmol). To this mixture was added dropwise with stirring a solution of $Li[N(SiMe_3)_2]$ (0.0430 g, 0.257 mmol) in THF (5 mL), resulting in a dark purple solution. After stirring at ambient temperature for 4 h, the solution was filtered through diatomaceous earth, and the resulting filtrate was dried under reduced pressure. The resulting residue was washed with hexanes (3 × 5 mL) and dried under reduced pressure for 12 h. Cold MeOH (10 mL) was then added at −78 ^oC and this solution was treated with a solution of $Na(BPh_4)$ (0.152 g, 0.444 mmol) in MeOH (5 mL) at −78 ^oC, resulting in an immediate precipitate. The solid was collected on a nylon filter (0.22 µm) and then washed with successive aliquots of MeOH (3 × 5 mL) and diethyl ether (3 × 5 mL), giving a gray filtrate and bright purple powder. Slow vapor diffusion of diethyl ether into an acetonitrile solution of the powder at ambient temperature gave **5**·2.9MeCN as long, purple plates suitable for single-crystal X-ray diffraction. The obtained crystals were dried under reduced pressure at ambient temperature for 2 h in order to remove the solvent molecules, yielding **5** (0.0734 g, 27%) as a dark yellow crystalline solid. Anal. Calcd. for $C_{144.8}H_{130.2}Cr_2N_{13.4}B_3$: C, 79.2; H, 5.98; N, 8.55%. Found: C, 79.0; H, 6.14; N, 8.31%. Absorption spectrum (MeCN): λ_{max} = 558 nm.

$[(TPyA)_2Mn_2(^{NMePh}L^{3-\bullet})](BAr^F{}_4)\cdot1.04[(Cp)Fe(C_6Me_6)](BAr^F{}_4)\cdot0.37THF$ (6). $(Cp)Fe(C_6Me_6)$ (0.0165 g, 0.0583 mmol) was dissolved in THF (1 mL). A portion of this solution (0.5 mL) was added dropwise at −78 ^oC to a stirring solution of **2** (0.0835 g, 0.0286 mmol) in THF (2 mL), resulting in a red-brown solution. After stirring for 20 minutes, this solution was treated with cold (−78 ^oC) hexanes (20 mL), resulting in the precipitation of a dark red-brown microcrystalline solid. The solid was collected on a nylon membrane filter (0.22 µm), washed with successive aliquots of cold hexanes (3 × 2 mL) and pentane (3 × 2 mL), and allowed to dry on the filter to yield **6** (0.0710 g, 76%). Anal. Calcd. for $C_{154.3}H_{117.3}N_{12}B_{2.04}F_{48.9}Mn_2Fe_{1.04}O_{0.4}$: C, 56.8; H, 3.62; N, 5.15 %. Found: C, 56.7; H, 3.69; N, 5.18 %. Absorption spectrum (THF): λ_{max} = 567 nm.

$[(TPyA)_2Fe_2(^{NMePh}L^{3-\bullet})](BAr^F{}_4)\cdot1.06[(Cp)Fe(C_6Me_6)](BAr^F{}_4)\cdot1.6THF$ (7). $(Cp)Fe(C_6Me_6)$ (0.0215 g, 0.0759 mmol) was dissolved in 1 mL THF. A portion of this solution (0.5 mL) was added dropwise at −78 ^oC to a stirring solution of **3** (0.1068 g, 0.0367 mmol) in THF (2 mL), resulting in a red

solution. After stirring for 20 minutes, this solution was treated with cold (−78 °C) hexanes (20 mL), resulting in the precipitation of a dark red microcrystalline solid. The solid was collected on a nylon membrane filter (0.22 µm), washed with successive aliquots of cold hexanes (3 × 2 mL) and pentane (3 × 2 mL), and allowed to dry on the filter to yield **5** (0.0528 g, 43%). Anal. Calcd. for $C_{160.3}H_{127.9}N_{12}B_{2.06}F_{49.4}Fe_{3.06}O_{1.6}$: C, 57.0; H, 3.81; N, 4.97 %. Found: C, 56.8; H, 4.17; N, 5.14 %. Absorption spectrum (THF): λ_{max} = 560 nm.

$[(TPyA)_2Fe_2(^{NMePh}L^{3-\bullet})](BAr^F_4)\cdot 1.35[(C_5Me_5)_2Co](BAr^F_4)$ (7) $(C_5Me_5)_2Co$ (0.0155 g, 0.0471 mmol) was added as a solid at −78 °C to a stirring solution of **3** (0.128 g, 0.0439 mmol) in THF (3 mL), resulting in a red solution. After stirring for 20 minutes, this solution was treated with cold (−78 °C) hexanes (20 mL), resulting in the precipitation of a dark red microcrystalline solid. The solid was collected on a nylon membrane filter (0.22 µm), washed with successive aliquots of cold hexanes (3 × 2 mL) and pentane (3 × 2 mL), and allowed to dry on the filter to yield **7′** (0.0830 g, 52%). Anal. Calcd. for $C_{172.2}H_{134.7}N_{12}B_{2.35}F_{56.4}Fe_2Co_{1.35}$: C, 56.5; H, 3.71; N, 4.59%. Found: C, 56.4; H, 3.96; N, 4.76%. Absorption spectrum (THF): λ_{max} = 564 nm.

$[(TPyA)_2Co_2(^{NMePh}L^{3-\bullet})](BAr^F_4)\cdot 0.94[(Cp)Fe(C_6Me_6)](BAr^F_4)\cdot 1.2THF$ (8). $(Cp)Fe(C_6Me_6)$ (0.0228 g, 0.0805 mmol) was dissolved in 1 mL THF. A portion of this solution (0.5 mL) was added dropwise at −78 °C to a stirring solution of **4** (0.110 g, 0.0375 mmol) in THF (2 mL), resulting in a dark purple solution. After stirring for 20 minutes, this solution was treated with cold (−78 °C) hexanes (20 mL), resulting in the precipitation of a dark purple microcrystalline solid. The solid was collected on a nylon membrane filter (0.22 µm), washed with successive aliquots of cold hexanes (3 × 2 mL) and pentane (3 × 2 mL), and allowed to dry on the filter to yield **8** (0.0442 g, 36%). Anal. Calcd. for $C_{154.3}H_{117.3}N_{12}B_{2.04}F_{48.9}Mn_2Fe_{1.04}$: C, 56.8; H, 3.62; N, 5.15 %. Found: C, 56.7; H, 3.69; N, 5.18 %. Absorption spectrum (THF): λ_{max} = 590 nm.

X-ray Structure Determination. Single crystals of **1**, **2**·0.4THF, **3**·2.5THF, **4**·2.5THF, and **5**·2.9MeCN were coated with deoxygenated Paratone-N oil, mounted on a MicroMounts™ rod, and frozen under a stream of N_2 during data collection. Crystallographic data were collected at 100 K using a Bruker Kappa Apex II diffractometer equipped with an APEX-II detector, a CuKα 1 µS microfocus source, and MX Optics. Raw data were integrated and corrected for Lorentz and polarization effects with SAINT v8.27B.[9] Absorption corrections were applied using SADABS. Space group assignments were determined by examination of systematic absences, E-statistics, and successive refinement of the structure. Structures were solved using direct methods in SHELXT and further refined with SHELXL-2014[10] operated with the OLEX2 interface.[11] All hydrogen atoms were placed at calculated positions using suitable riding models and refined using isotropic displacement parameters derived from their parent atoms. Thermal parameters were refined anisotropically for all non-hydrogen atoms. Crystallographic data for these compounds at 100 K are given in Table 1. Significant disorder of the *o*-tolyl groups on the bridging ligand was modeled with partial occupancies; however, some disorder that did not improve the final refinement of the solution was placed at one position with a full occupancy. At 100 K, all solvent molecules in **1**, **2**·0.4THF, and **4**·2.5THF, one THF molecule in **3**·2.5THF, and 4.6 MeCN molecules in **5** were severely disordered and could not be modeled properly. These species were therefore treated as a diffuse contribution to the overall scattering without specific atom positions using the solvent masking procedure implemented in OLEX2. These molecules were included in the final molecular formula.

Magnetic Measurements. All samples were prepared and manipulated with the rigorous exclusion of dioxygen under a dinitrogen atmosphere. Magnetic measurements of **2-8** were performed on

polycrystalline samples sealed in a 2 mL polyethylene bag. All data were collected using a Quantum Design MPMS-XL SQUID magnetometer from 1.8 to 300 K at applied dc fields ranging from 0 to +7 T. Dc susceptibility data were corrected for diamagnetic contributions from the sample holder and for the core diamagnetism of each sample (estimated using Pascal's constants[12]). *M* vs *H* curves, constructed from data collected from 0 to 3 T at 100 K, confirmed the absence of significant ferromagnetic impurities in all samples. Experimental errors for magnetic exchange coupling in compounds **5-8** were determined by averaging simulations of two independently prepared samples.

Mössbauer Measurements. Zero-field iron-57 Mössbauer spectra were obtained at 80 K with a constant acceleration spectrometer and a cobalt-57 rhodium source. Prior to measurements, the spectrometer was calibrated at 295 K with α-iron foil. Samples were prepared in a dinitrogen-filled glovebox with crystals of **3** and **7′** that were inserted into the cryostat prior to the measurement. The samples contained approximately 80 mg/cm^2 of compound. All spectra were analyzed using the WMOSS Mössbauer Spectral Analysis Software (www.wmoss.org). Fits to a minor doublet in the spectrum of **3** at 80 K gave an isomer shift of δ = 0.08(1) mm/s and a quadrupole splitting of ΔE_Q = 0.73(3) mm/s, consistent with a small amount of high-spin Fe^{III}-containing impurity in the sample.

Other Physical Measurements. Elemental analyses of all compounds were performed by Midwest Microlab (Indianapolis, IN). Infrared spectra were recorded on a Bruker Alpha FTIR spectrometer equipped with an attenuated total reflectance accessory. ^{1}H NMR spectra were collected at 500 MHz on a Varian Inova 500 system at 298 K. UV/Vis/NIR spectra were obtained using either a Varian Cary 60 spectrophotometer or a Varian Cary 5000 spectrophotometer. Cyclic voltammetry measurements were carried out in a standard one-compartment cell under nitrogen, equipped with a glassy carbon working electrode, a platinum wire as counter electrode, and a silver wire as reference electrode using a CHI 760c potentiostat. Analyte solutions were prepared with 0.05 M solutions of $Na(BAr^F_4)$ in THF (**2-4**) or 0.1 M solutions of $(NBu_4)PF_6$ in MeCN (**5**). Ferrocene (Cp_2Fe) was employed as an internal standard and all potentials were referenced to the $[Cp_2Fe]^{0/1+}$ couple.

Table S1. Crystallographic data for compounds

	**1**	**2**·0.4THF	**3**·2.5THF	**4**·2.5THF	**5**·2.9MeCN
formula	$C_{174}H_{158}B_4Cr_2N_{16}$	$C_{135.6}H_{93.2}B_2F_{48}Mn_2N_{12}O_{0.4}$	$C_{144}H_{110}B_2F_{48}Fe_2N_{12}O_{2.5}$	$C_{144}H_{110}B_2Co_2F_{48}N_{12}O_{2.5}$	$C_{301.2}H_{277.8}B_6Cr_4N_{32.6}$
fw, g/mol	2620.46	2911.67	3093.73	3099.91	4627.10
T, K	100	100	100	100	100
space group	$P\,-1$	$P\,-1$	$P\,-1$	$P\,-1$	$P\,-1$
Z	1	1	1	1	1
a, Å	15.3682(7)	15.0589(5)	15.17(1)	14.8476(4)	15.1700(6)
b, Å	15.9908(7)	15.8311(5)	15.92(1)	15.9017(4)	15.9112(6)
c, Å	17.592(1)	18.4096	18.40(1)	18.2100(5)	30.342(1)
α, deg	72.813(3)	75.382(2)	75.334(2)	75.560(2)	90.924(2)
β, deg	69.832(4)	67.031(2)	66.781(2)	67.648(2)	97.350(2)
γ, deg	68.859(3)	68.819(2)	68.243(2)	68.920(2)	115.288(2)
V, Å^3	3712.1(3)	3736.0(2)	3763.5(4)	3676.4(2)	6546.9(4)
d_{calc}, g/cm^3	1.081	1.294	1.333	1.312	1.126
R_1 $(I > 2\sigma(I))^a$	8.22	5.99	6.31	6.68	8.12
wR_2 $(all)^b$	22.22	18.31	16.64	19.36	23.95
GoF	0.944	1.080	1.073	1.066	1.008

$^aR_1 = \Sigma||F_0| - |F_C||/\Sigma|F_0|$. $^bwR_2 = [\Sigma w(F_0^2 - F_C^2)^2/\Sigma w(F_0^2)^2]^{1/2}$.

Table S2. Selected interatomic distances (Å) and angles (°) for compounds[a]

	**1**	**2**·0.4THF	**3**·2.5THF	**4**·2.5THF	**5**·2.9MeCN	
M-N3	2.119(3)	2.361(3)	2.288(3)	2.261(3)	2.132(3)	2.127(4)
M-N4	2.112(3)	2.271(3)	2.201(3)	2.161(3)	2.106(3)	2.098(4)
M-N5	2.070(3)	2.271(3)	2.188(3)	2.152(3)	2.063(3)	2.075(3)
M-N6	2.142(3)	2.346(3)	2.323(3)	2.265(3)	2.166(3)	2.180(5)
**M-N_{TPyA}**	**2.111(6)**	**2.312(6)**	**2.250(6)**	**2.210(6)**	**2.117(6)**	**2.120(6)**
M-N1	2.034(3)	2.163(2)	2.131(2)	2.116(3)	1.988(3)	1.982(4)
M-N2	2.025(3)	2.137(2)	2.084(2)	2.034(3)	2.002(3)	1.994(4)
**M-N_L**	**2.030(4)**	**2.150(3)**	**2.108(3)**	**2.075(4)**	**1.995(4)**	**1.988(6)**
C1-C2	1.398(5)	1.404(4)	1.402(4)	1.408(5)	1.398(5)	1.406(6)
C2-C3	1.475(5)	1.499(4)	1.497(4)	1.495(5)	1.446(5)	1.443(7)
C3-C1A	1.406(4)	1.383(4)	1.381(4)	1.375(5)	1.397(5)	1.412(6)
**C-C**	**1.426(8)**	**1.429(7)**	**1.427(7)**	**1.426(9)**	**1.414(9)**	**1.42(1)**
N1-C2	1.337(4)	1.315(4)	1.315(4)	1.312(4)	1.362(5)	1.367(5)
N2-C3	1.333(5)	1.340(4)	1.348(4)	1.355(4)	1.366(5)	1.357(6)
**N-C**	**1.335(6)**	**1.328(6)**	**1.332(6)**	**1.334(6)**	**1.364(7)**	**1.362(8)**
N_L-M-N_L	79.3(1)	76.83(8)	77.83(9)	78.8(1)	79.7(1)	80.5(2)

[a]See Figures 1 and 2 for numbering scheme.

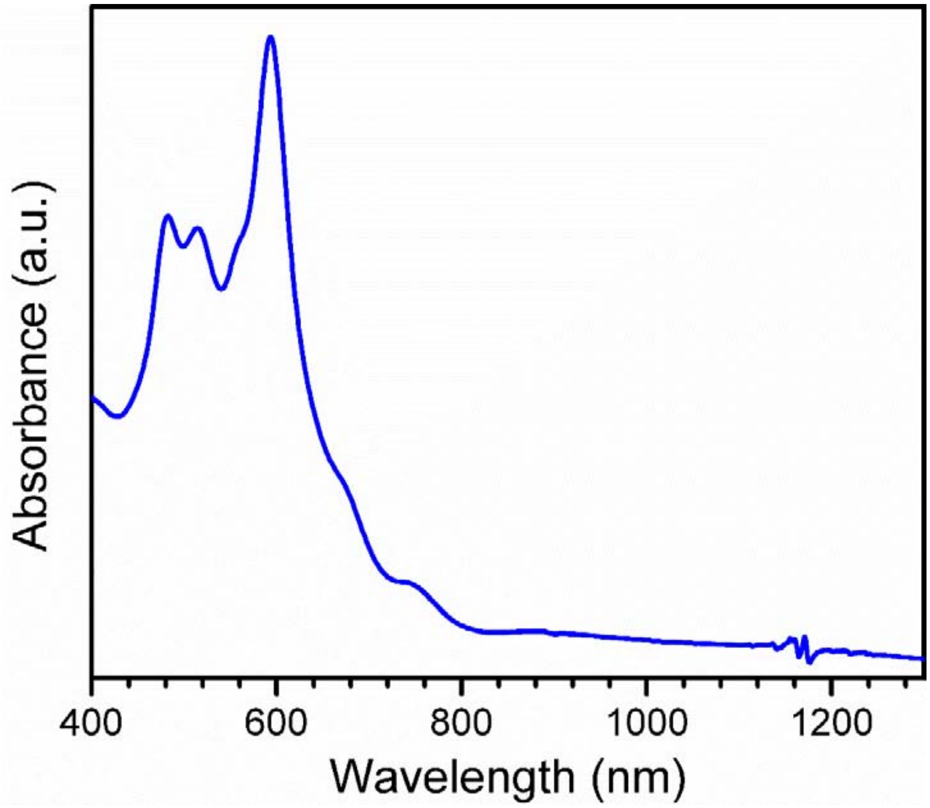

Figure S1 | UV/Vis spectrum of **5** in MeCN at ambient temperature.

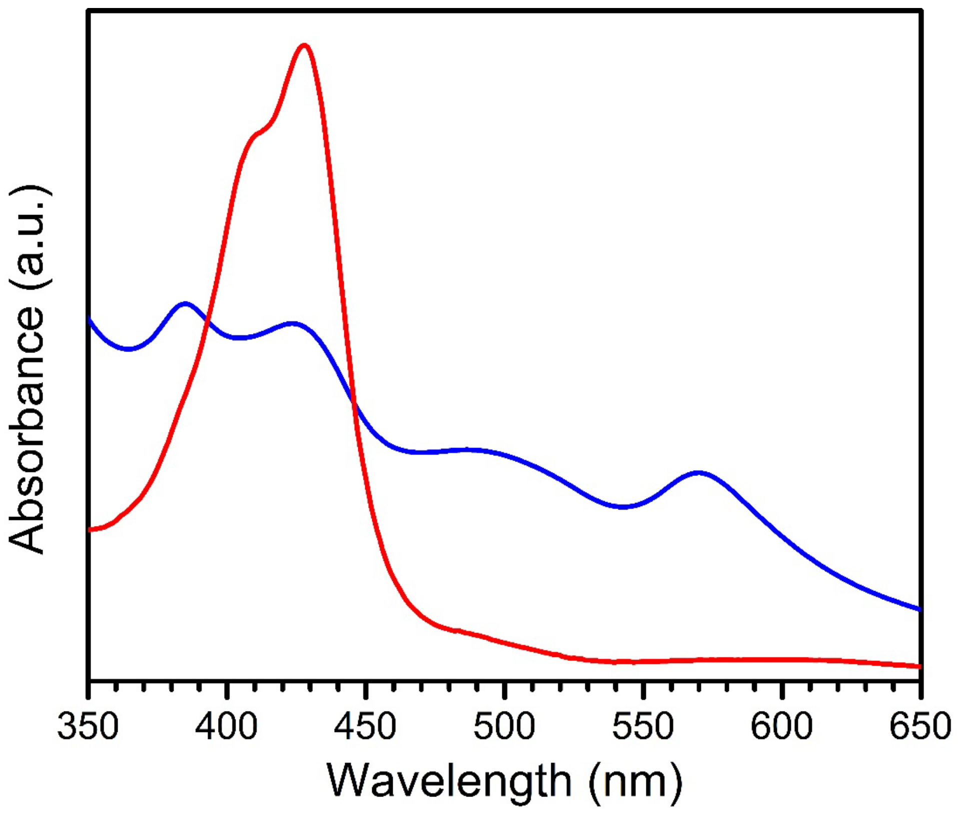

Figure S2 | UV/Vis spectrum of **2** (red) and **6** (blue) in THF at ambient temperature.

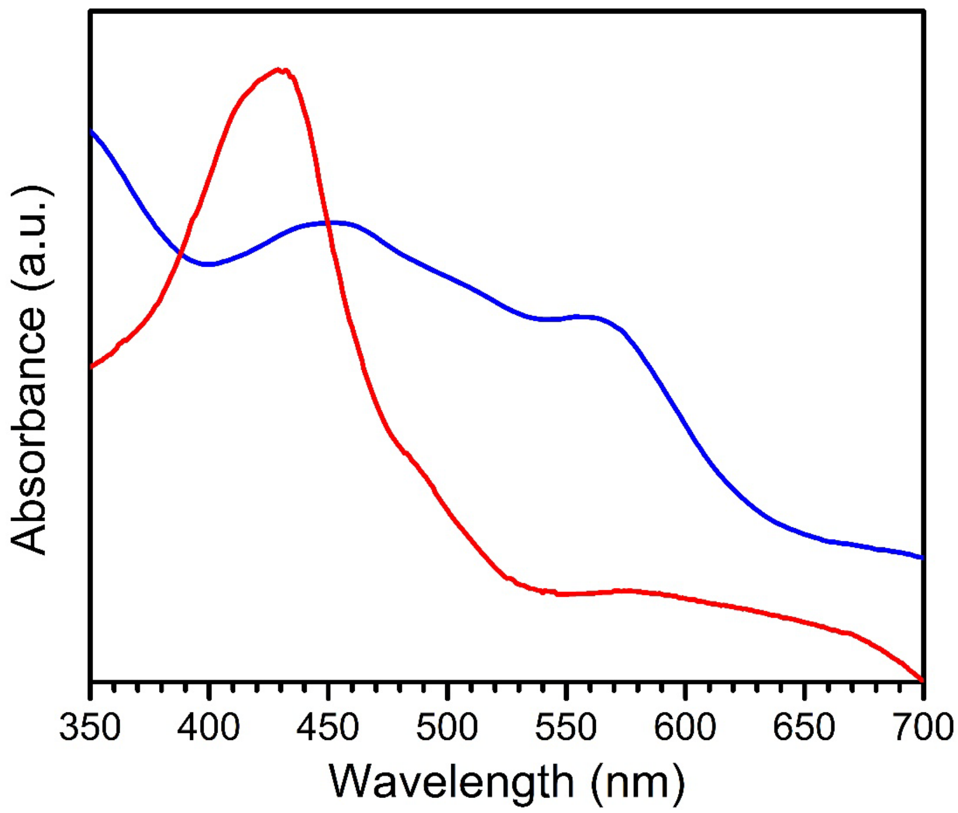

Figure S3 | UV/Vis spectrum of **3** (red) and **7** (blue) in THF at ambient temperature.

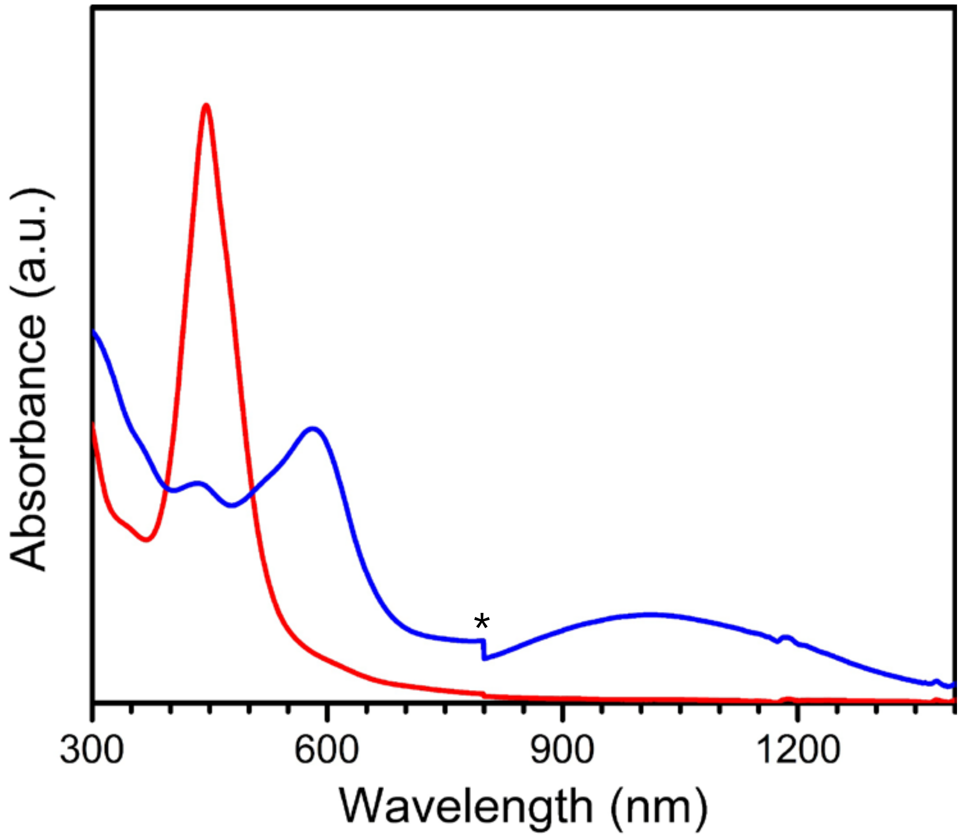

Figure S4 | UV/Vis/NIR spectra of **4** (red) and **8** (blue) in THF at ambient temperature. The asterisk denotes an instrument derived artifact.

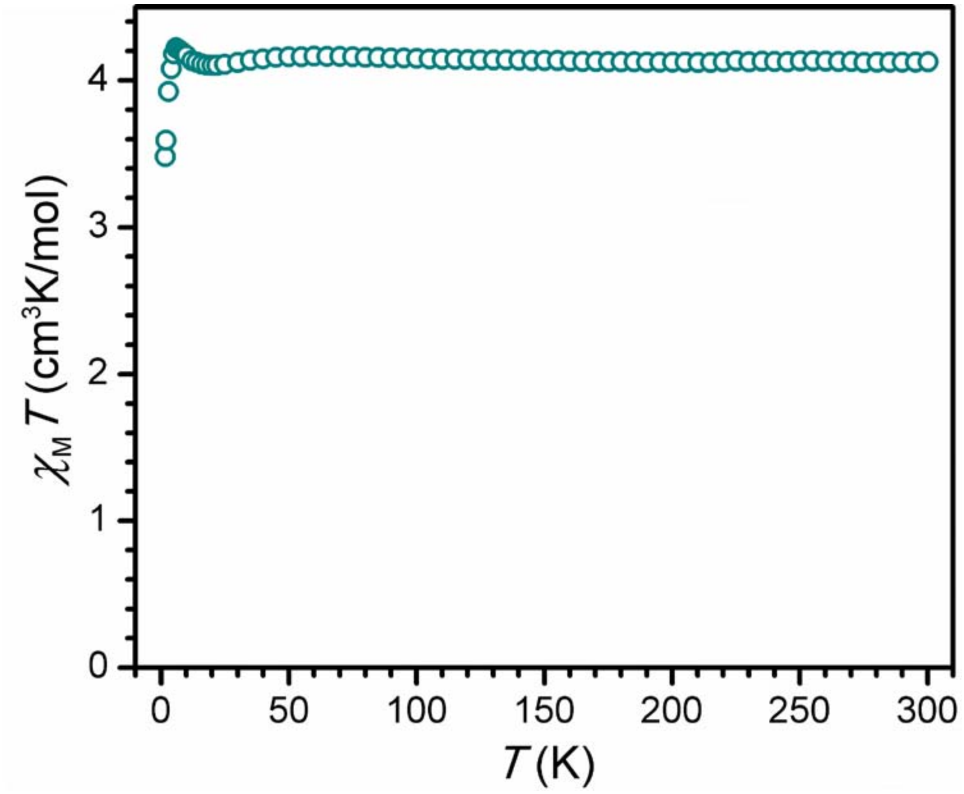

Figure S5. Variable-temperature dc magnetic susceptibility of **4** collected at a field of 0.1 T. Note the pronounced upturn below 20K indicative of weak, ferromagnetic coupling.

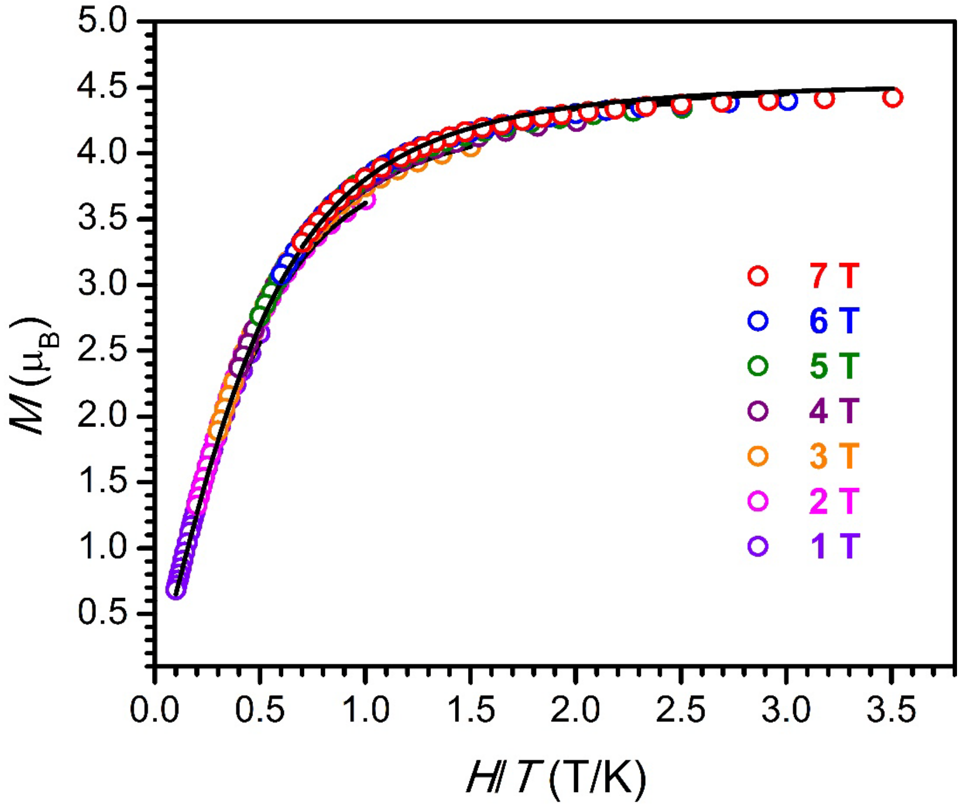

Figure S6 | Low-temperature magnetization data for **5** at selected fields. Black lines indicate fits to data.

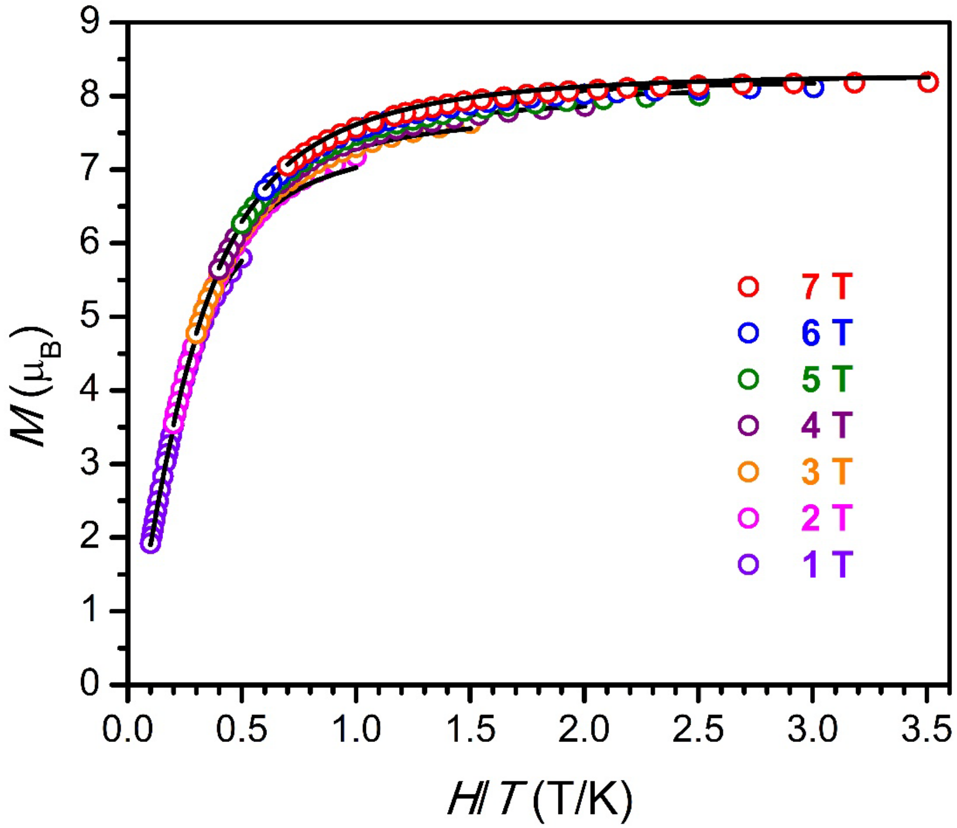

Figure S7 | Low-temperature magnetization data for **6** at selected fields. Black lines indicate fits to data.

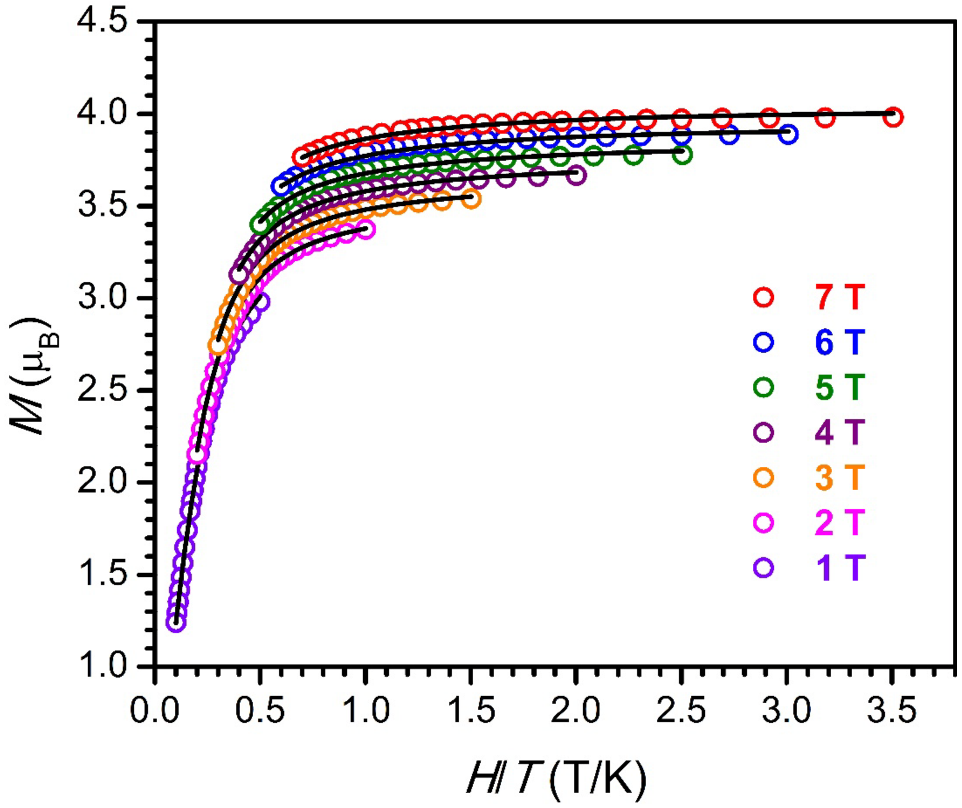

Figure S8 | Low-temperature magnetization data for **7** at selected fields. Black lines indicate fits to data.

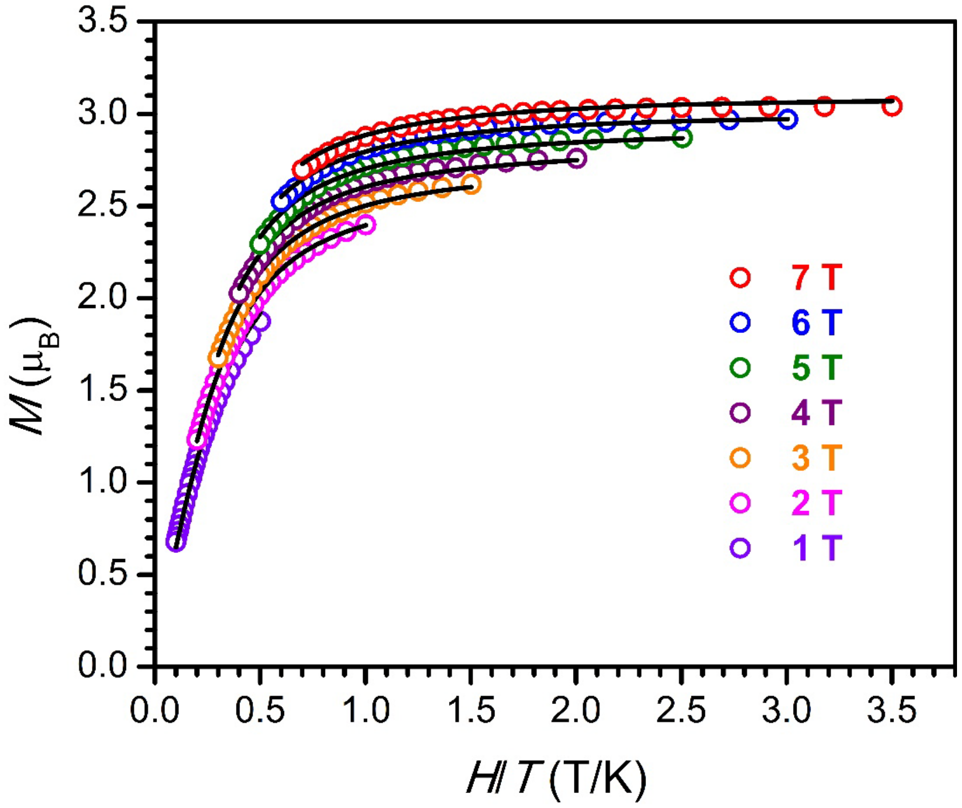

Figure S9 | Low-temperature magnetization data for **8** at selected fields. Black lines indicate fits to data.

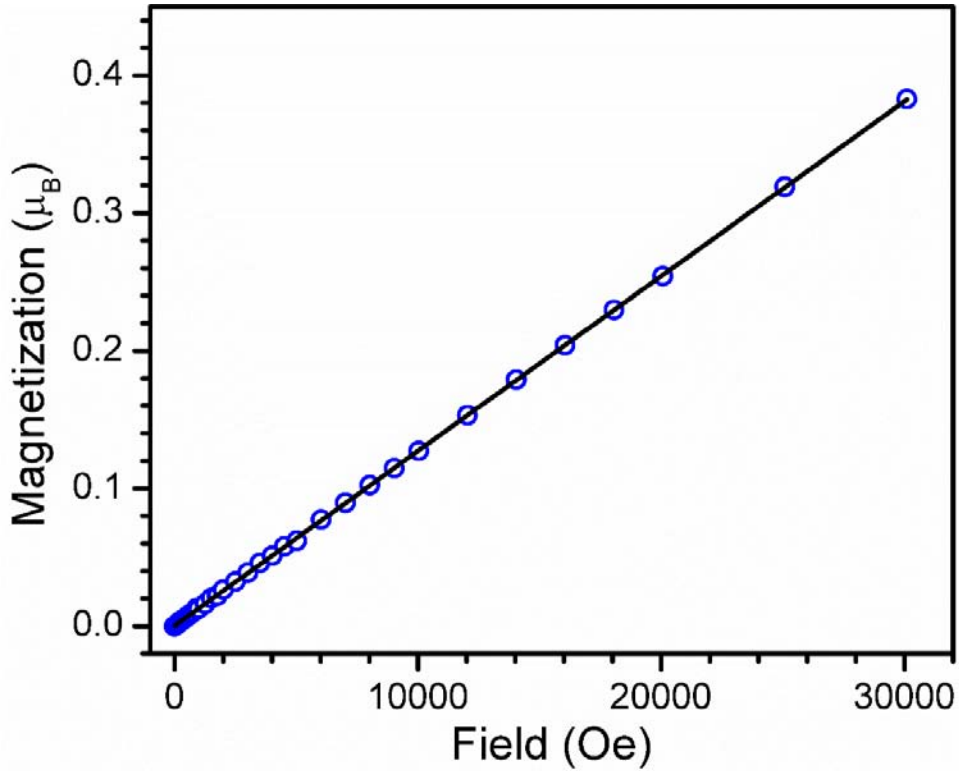

Figure S10 | Variable-field magnetization of **2** at 100K. Black line indicates a linear fit.

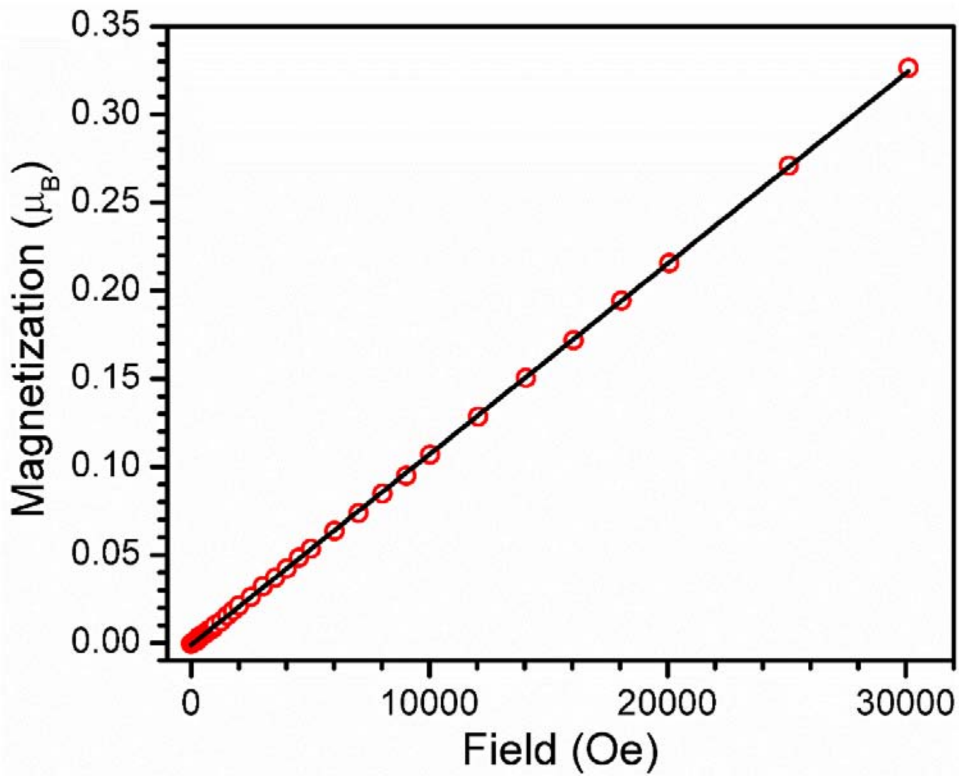

Figure S11 | Variable-field magnetization of **3** at 100K. Black line indicates a linear fit.

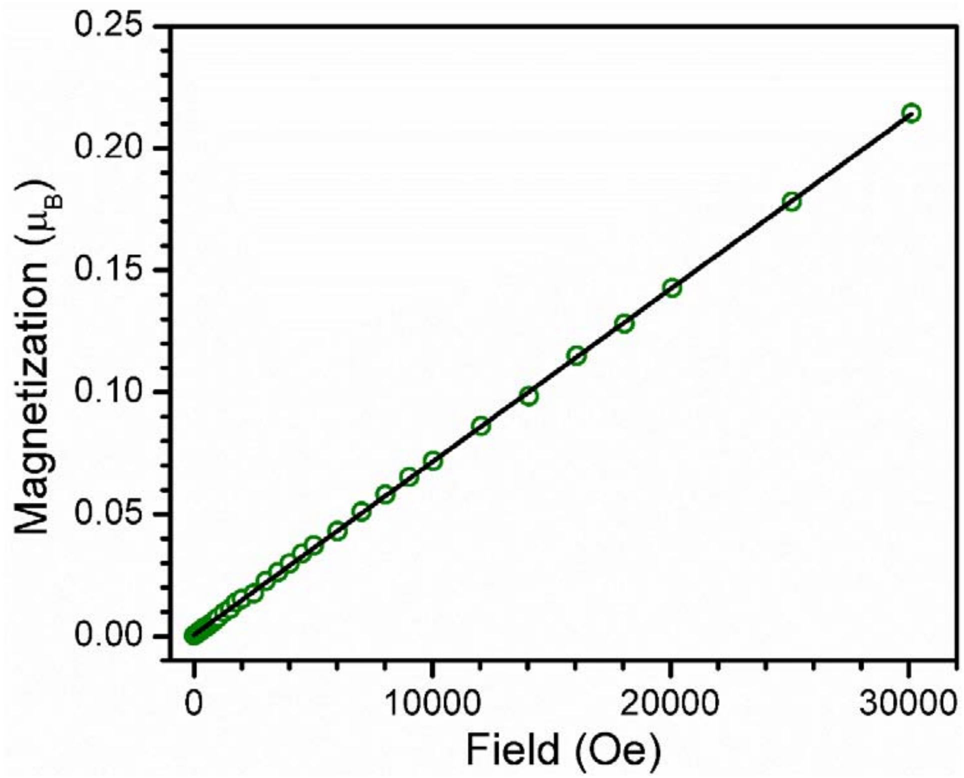

Figure S12 | Variable-field magnetization of **4** at 100K. Black line indicates a linear fit.

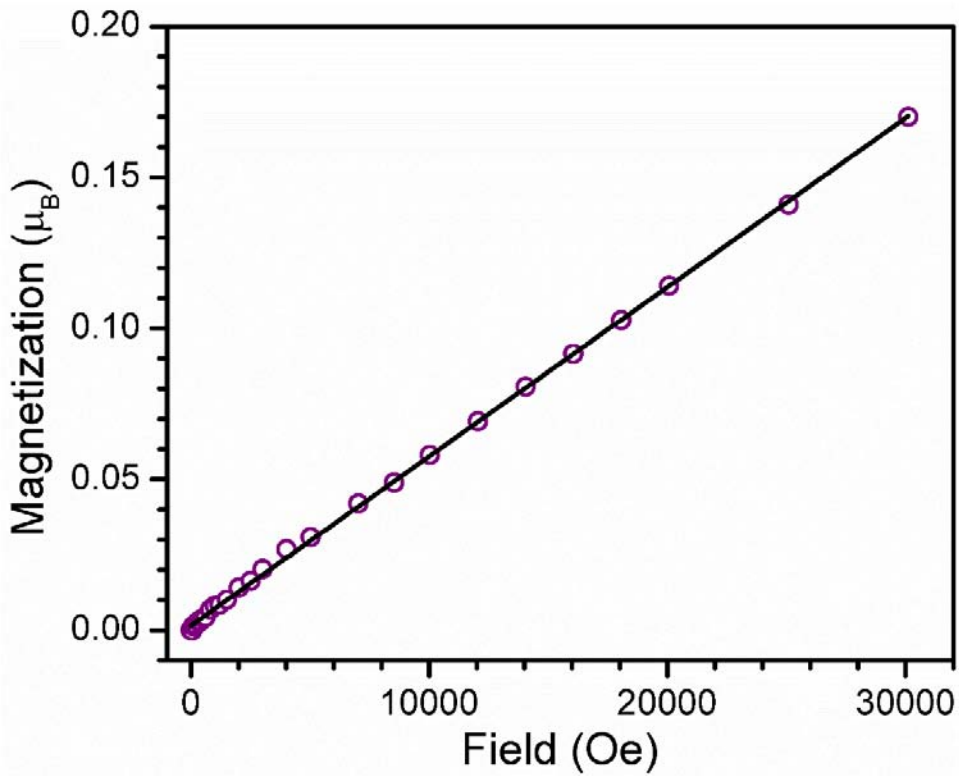

Figure S13 | Variable-field magnetization of **5** at 100K. Black line indicates a linear fit.

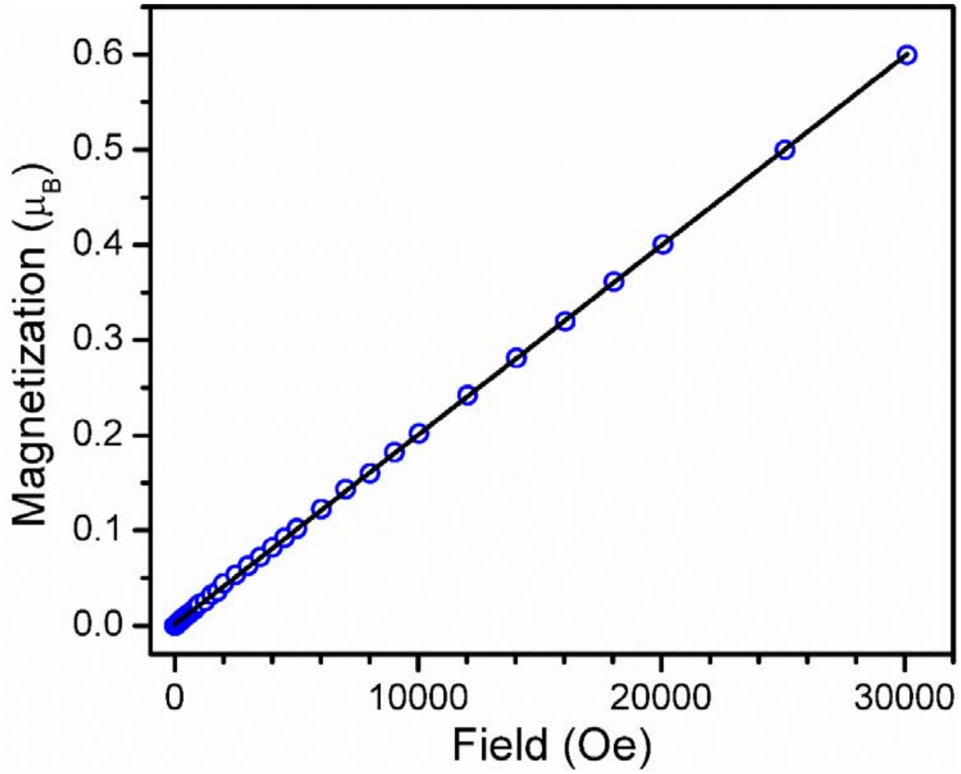

Figure S14 | Variable-field magnetization of **6** at 100K. Black line indicates a linear fit.

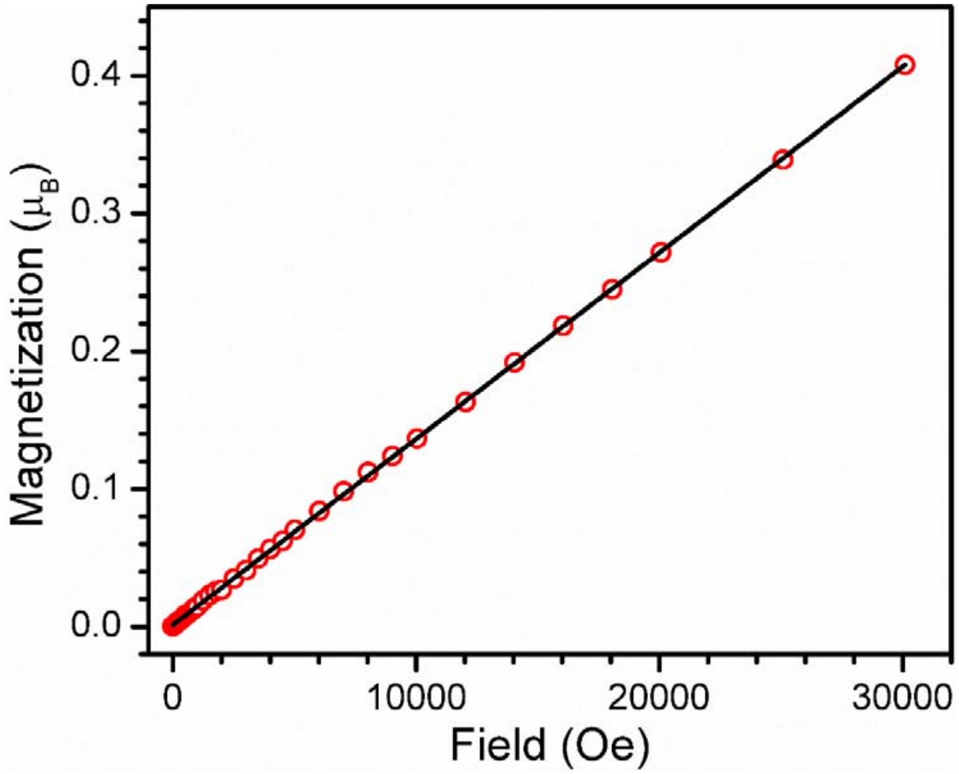

Figure S15 | Variable-field magnetization of **7** at 100K. Black line indicates a linear fit.

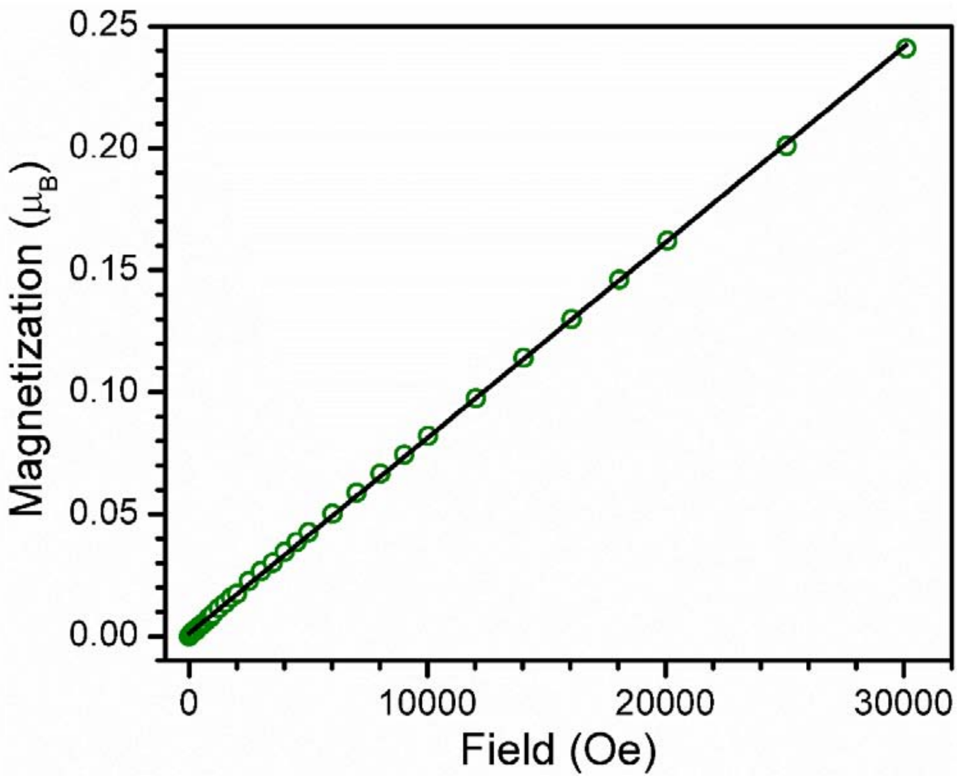

Figure S16 | Variable-field magnetization of **2** at 100K. Black line indicates a linear fit.

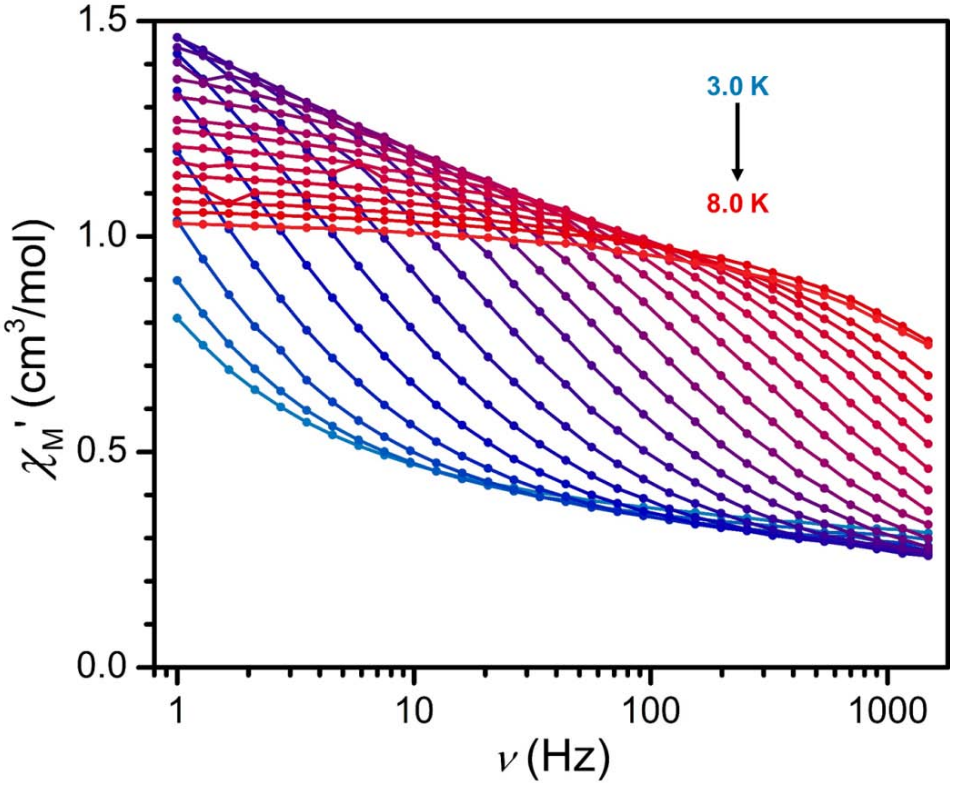

Figure S17 | Plot of in-phase ac magnetic susceptibility for **7**.

References

(1) W.E. Buschmann and J. S. Miller, *Chem. Eur. J.*, 1998, **4**, 1731.

(2) G. J. P. Britovsek, J. England and A. J. P. White, *Inorg. Chem.*, 2005, **44**, 8125.

(3) E. H. Amonoo-Neizer, R. A. Shaw, D. O. Skovlin and B. C. Smith, *Inorg. Synth.*, 1966, **8**, 19.

(4) J. G. Park, I.-R. Jeon and T. D. Harris, *Inorg. Chem.*, 2014, **54**, 359.

(5) R. Jordan, R. Lapointe, C. Bajgur, S. Echols and R. Willett, *J. Am. Chem. Soc.*, 1987, **109**, 4111.

(6) A. N. Nesmeyanov, N. A. Vol'kenau and I. N. Bolesova, *Tetrahedron Lett.*, 1963, **4**, 1725.

(7) J.-R. Hamon, D. Astruc and P. Michaud, *J. Am. Chem. Soc.*, 1981, **103**, 758.

(8) (*a*) T. Wenderski, K. M. Light, D. Ogrin, S. G. Bott and C. J. Harlan, *Tetrahedron Lett.*, 2004, **45**, 6851. (*b*) D. M. Khramov, A. J. Boydston and C. W. Bielawski, *Org. Lett.*, 2006, **8**, 1831.

(9) *SAINT v8.27B Software for the Integration of CCD Detector System*; Bruker AXS Inc., Madison, WI, USA, 2007.

(10) G. M. Sheldrick, *Acta Crystallogr. Sect. A Found. Crystallogr.*, 2008, **64**, 112.

(11) O. V. Dolomanov, L. J. Bourhis, R. J. Gildea, J. A. K. Howard and H. Puschmann, *J. Appl. Crystallogr.*, 2009, **42**, 339.

(12) G. A. Bain and J. F. Berry, *J. Chem. Ed.*, 2008, **85**, 532.
